# Three-pathway combination for glutathione biosynthesis in *Saccharomyces cerevisiae*

**DOI:** 10.1186/s12934-015-0327-0

**Published:** 2015-09-16

**Authors:** Liang Tang, Weiwei Wang, Wenlong Zhou, Kai Cheng, Yan Yang, Minzhi Liu, Kedi Cheng, Wei Wang

**Affiliations:** State Key Laboratory of Bioactive Substance and Function of Natural Medicines, Institute of Materia Medica, Peking Union Medical College and Chinese Academy of Medical Sciences, 1 Xian Nong Tan St., 100050 Beijing, China; Key Laboratory of Biosynthesis of Natural Products of National Health and Family Planning Commission, Institute of Materia Medica, Peking Union Medical College and Chinese Academy of Medical Sciences, 1 Xian Nong Tan St., 100050 Beijing, China; College of Life Science, Qufu Normal University, 273165 Qufu, Shandong China

**Keywords:** Glutathione, Three-pathway combination, Combinatorial biosynthesis, *Saccharomyces cerevisiae*

## Abstract

**Background:**

Glutathione (GSH), a pivotal non-protein thiol, can be biosynthesized through three pathways in different organisms: (1) two consecutive enzymatic reactions catalyzed by γ-glutamylcysteine synthetase (Gsh1 or GshA) and glutathione synthetase (Gsh2 or GshB); (2) a bifunctional γ-glutamylcysteine synthetase/glutathione synthetase (GshF); (3) an alternative condensation of γ-glutamyl phosphate synthesized by γ-glutamyl kinase (Pro1 or ProB) with cysteine to form γ-glutamylcysteine which was further conjugated to glycine by glutathione synthetase. The Gsh1 and Gsh2 of conventional GSH biosynthetic pathway or the bifunctional GshF reported previously have been independently modulated for GSH production. This study developed a novel three-pathway combination method to improve GSH production in *Saccharomyces cerevisiae.*

**Results:**

A bifunctional enzyme GshF of *Actinobacillus pleuropneumoniae* was functionally expressed in *S. cerevisiae* and Pro1 in proline biosynthetic pathway was exploited for improving GSH yield. Moreover, two fusion proteins Gsh2-Gsh1 and Pro1-GshB were constructed to increase the two-step coupling efficiency of GSH synthesis by mimicking the native domain fusion of GshF. The engineered strain W303-1b/FGP with three biosynthetic pathways presented the highest GSH concentration (216.50 mg/L) and GSH production of W303-1b/FGP was further improved by 61.37 % when amino acid precursors (5 mM glutamic acid, 5 mM cysteine and 5 mM glycine) were fed in shake flask cultures. In batch culture process, the recombinant strain W303-1b/FGP also kept high efficiency in GSH production and reached an intracellular GSH content of 2.27 % after 24-h fermentation.

**Conclusions:**

The engineered strains harbouring three GSH pathways displayed higher GSH producing capacity than those with individually modulated pathways. Three-pathway combinatorial biosynthesis of GSH promises more effective industrial production of GSH using *S. cerevisiae*.

**Electronic supplementary material:**

The online version of this article (doi:10.1186/s12934-015-0327-0) contains supplementary material, which is available to authorized users.

## Background

Glutathione (γ-l-glutamyl-l-cysteinylglycine, GSH) is an intracellular redox-active tripeptide thiol found in living organisms [1, 2]. The free sulfhydryl moiety of cysteine residue contributes to a wide variety of biological activities, such as anti-oxidization [3], detoxification [4, 5] and immune regulation [6], etc. GSH has been widely used in pharmaceutical, food and cosmetic industries.

**Fig. 1:**
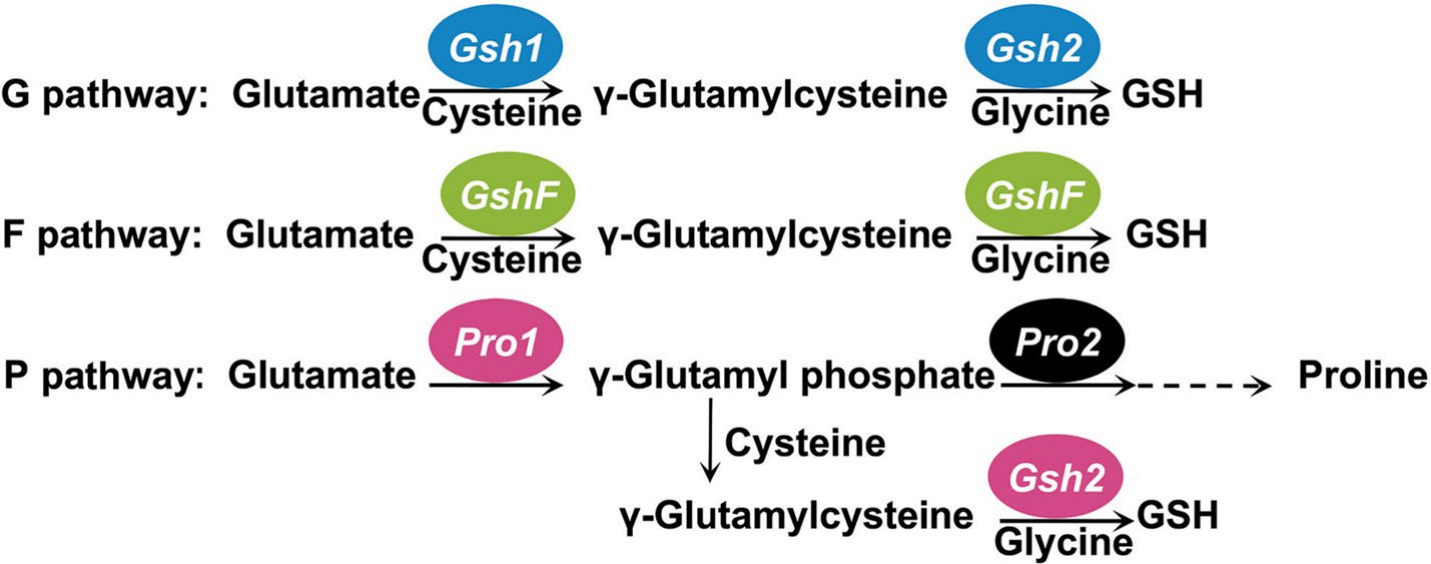
Biosynthetic pathways of GSH. G pathway, Gsh1 catalyzes the reaction between glutamic acid and cysteine to form γ-GC, which is subsequently conjugated to glycine by Gsh2 to synthesize GSH. F pathway, GshF accounts for Gsh1 and Gsh2 activities and carries out complete synthesis of GSH. P pathway, Pro1 converts glutamate to γ-glutamyl phosphate, which reacts with cysteine to generate γ-GC, and then Gsh2 adds glycine to γ-GC to form GSH

GSH is primarily synthesized intracellularly through two consecutive reactions catalyzed by γ-glutamylcysteine synthetase (Gsh1 in eukaryotes and GshA in prokaryotes, encoded by *GSH1* and *gshA*, respectively) and glutathione synthetase (Gsh2 in eukaryotes and GshB in prokaryotes, encoded by *GSH2* and *gshB*, respectively). Gsh1 catalyzes the formation of γ-glutamylcysteine (γ-GC), which is subsequently conjugated to glycine by Gsh2 to form GSH. The activity of Gsh1 could be physiologically feedback-inhibited by GSH to avoid over-accumulation of GSH [7]. However, many organisms without Gsh1 or Gsh2 were reported to have the ability to produce GSH, which suggests that they may have a different pathway for generation of GSH. Recently, a novel bifunctional enzyme GshF (encoded by *gshF*) found in some Gram-positive bacteria such as: *Listeria monocytogenes*, *Streptococcus agalactiae* and *Pasteurella multocida*, was able to perform complete synthesis of GSH [8–11]. In other studies, compensatory pathway for GSH synthesis was characterized in yeast and *Escherichia coli* that lack Gsh1 and GshA [12, 13]. γ-glutamyl kinase (GK) encoded by *PRO1* in eukaryotes or *proB* in prokaryotes, the first enzyme in proline biosynthetic pathway, catalyzes the formation of intermediate γ-glutamyl phosphate, which if accumulated could partially react with cysteine to generate γ-GC, and then Gsh2 adds glycine to γ-GC to form GSH. Different biosynthetic pathways of GSH are as shown in Fig. 1 and referred as G pathway, F pathway and P pathway.

To date, GSH is mainly produced via fermentation. Yeasts, usually *S. cerevisiae* and *Candida utilis*, are more commonly used in industrial scale GSH production. GSH contents of the wild type (wt) yeasts are typically in the range of 0.1–1 %. The most successful strategy toward obtaining GSH overproducing strains was classical selection which resulted in GSH contents of 3–5 % [14]. Along with the identification and characterization of genes involved in GSH metabolism, many genetic engineering strategy studies have been conducted in an effort to increase GSH production using various model microorganisms. The reported GSH contents in genetically engineering strains ranged from 1 to 2 % in the absence of amino acid precursors [15–22]. Genetic manipulation of the GSH biosynthetic pathway has been proved to be an effective method to produce GSH and provides a platform for further improvement. In previous reports, single modulation of G pathway and F pathway for GSH production could achieve GSH contents of 1.5–1.7 % [19, 22] (Table 1). Besides, many efforts have been focused on medium optimization and process control to enhance GSH production, in particular introduction of amino acid precursors during GSH fermentation [23, 24].

**Table 1:** An overview of literature on the production of GSH without addition of amino acid precursors by microorganisms

Host strain	Strategy	GSH content in mutant	GSH content in wild type	References
*C. utilis*	Classical selection	3–5 %	0.1–1 %	[14]
*S. cerevisiae*				
*S. cerevisiae*	pGSR2518-x containing *gshA* of *E. coli* B fused with a *S. cerevisiae* promoter fragment *P8* was used to transform *S. cerevisiae* YNN27	1.54 %	0.5 %	[17]
*S. cerevisiae*	A recombinant plasmid pGMF with *GSH1* from *S. cerevisiae* was introduced into *S. cerevisiae* YSF-31	1.31 %	0.87 %	[18]
*S. cerevisiae*	Plasmids, pδAUR-GCS and pδAUR-GS, containing *GSH1* and *GSH2* from *S. cerevisiae* were linearized and integrated at δ-sites with high copy numbers into the ribosomal DNA of *S. cerevisiae* YPH499	1.51 %	1.03 %	[19]
	Sulfate assimilation metabolism and GSH synthetic metabolism were combinatorially engineered in *S. cerevisiae* YPH499	1.83 %		
*S. cerevisiae*	*gshF* derived from *Streptococcus thermophilus* was integrated at a high copy number into the ribosomal DNA of *S. cerevisiae* BY4741	54.9 μM/g DCW (1.69 %)	11.7 μM/g DCW (0.36 %)	[22]
*P. pastoris*	An integrative expression vector, pGAPZHGSH, containing *GSH1* and *GSH2* from *S. cerevisiae* regulated by GAP promoter was transformed into *P. pastoris* GS115	0.92 g (GSH)/L		[16]
		94.98 g (DCW)/L (0.97 %)		
*P. pastoris*	An integrative expression vector, pGAPZHGSH, containing *GSH1* and *GSH2* from *S. cerevisiae* regulated by GAP promoter was transformed into *P. pastoris* GS115	<0.05 mM/g DCW (<1.54 %)	<0.02 mM/g DCW (<0.61 %)	[21]
	An integrative expression vector, pGAPZH-Lmgsh, containing *gshF* from *Listeria monocytogenes* regulated by GAP promoter was transformed into *P. pastoris* GS115	<0.04 mM/g DCW (<1.23 %)		

Although genetic manipulations of a single pathway have increased GSH production of engineered strains, discovering how to further improve GSH production is still a real challenge for us. In this study, we focused on direct modulation of the GSH biosynthetic pathway via a method of three-pathway combination. Two artificially recombinant enzymes glutathione synthetase-linker-γ-glutamylcysteine synthetase fusion protein (Gsh2-Gsh1) from *S. cerevisiae*, γ-glutamyl kinase-linker-glutathione synthetase hybrid (Pro1-GshB) from *S. cerevisiae* and *E. coli* and a resynthesized GshF of *Actinobacillus pleuropneumoniae* were introduced into *S. cerevisiae* genome via independent or combinatorial genetic integration. The overexpression of genes from different pathways and synthetic capacities of GSH of different engineered strains were investigated. The data demonstrated that three-pathway combination was superior to single-pathway for synthesis of GSH.

## Results

### Activity analysis of GshF from *A. pleuropneumoniae* expressed under the control of different promoters in *S. cerevisiae*

To examine the activity of codon-conformed *A. pleuropneumoniae* GshF in *S. cerevisiae* and evaluate effect of promoter strength on its activity, four δ DNA-mediated integrative expression vectors pδGAPg-gshF, pδGAP′g-gshF, pδGAL1g-gshF and pδPGK1g-gshF were constructed, in which the *gshF* gene was driven by glyceraldehyde-3-phosphate dehydrogenase promoter (GAP) of *S. cerevisiae*, GAP promoter from *Pichia pastoris* (GAP′), galactokinase promoter (GAL1) of *S. cerevisiae* and phosphoglycerate kinase promoter (PGK1) of *S. cerevisiae*, respectively. *S. cerevisiae* W303-1b cells were transformed with the linearized recombinant plasmids to obtain engineered strains W303-1b/F/GAP, W303-1b/F/GAP′, W303-1b/F/GAL1 and W303-1b/F/PGK1. The plasmids and strains constructed in the present study are listed in Table 2. GshF activities and promoter strengths could be determined by GSH concentrations of the engineered strains in shake flask cultures.

**Table 2:** Strains and plasmids used in this study

Strains or plasmids	Relevant properties	Source or references
Strains		
*E. coli* Trans1-T1	F^−^ *φ80*(*lacZ*) *ΔM15 ΔlacX74 hsdR*(rK^−^ mK^+^) *ΔrecA1398 endA1 tonA*	Our lab
*S. cerevisiae* W303-1b	*MATα* *ade2*-*1 leu2*-*3*,*112 his3*-*11*,*15 ura3*-*1 trp1*-*1*	Our lab
W303-1b/Δgsh1	W303-1b derivative with pΔgsh1h, *GSH1::HygB*	This study
W303-1b/Δgsh1/pro1	W303-1b derivative with pΔgsh1h-pro1, *GSH1::P*_*gap*_-*PRO1*-*T*_*pgk1*_	This study
W303-1b/Δgsh1/proB	W303-1b derivative with pΔgsh1h-proB, *GSH1::P*_*gap*_-*proB*-*T*_*pgk1*_	This study
W303-1b/F/GAP (W303-1b/F)^a^	W303-1b derivative with pδGAPg-gshF	This study
W303-1b/F/GAP′	W303-1b derivative with pδGAP′g-gshF	This study
W303-1b/F/PGK1	W303-1b derivative with pδPGK1g-gshF	This study
W303-1b/F/GAL1	W303-1b derivative with pδGAL1g-gshF	This study
W303-1b/Δgsh1/F	W303-1b/F derivative with pΔgsh1h, *GSH1::HygB*	This study
W303-1b/G	W303-1b derivative with pδGAPh-gsh2gsh1	This study
W303-1b/P	W303-1b derivative with pδGAPg-pro1gshB	This study
W303-1b/P′	W303-1b derivative with pδGAPg-proBgshB	This study
W303-1b/FF	W303-1b/F derivative with pδGAPg-gshF	This study
W303-1b/FG	W303-1b/F derivative with pδGAPh-gsh2gsh1	This study
W303-1b/FP	W303-1b/F derivative with pδGAPg-pro1gshB	This study
W303-1b/FGP	W303-1b/FG derivative with pδGAPg-pro1gshB	This study
Plasmids		
pG	pBluescript II KS(+) derivative with homologous region of *GSH1*	Our lab
pΔgsh1h	pBluescript II KS(+) derivative with homologous region of *GSH1*, HygB^r^	This study
pΔgsh1h-pro1	pG derivative with *P*_*gap*_, *PRO1*, *T*_*pgk1*_, HygB^r^	This study
pΔgsh1h-proB	pG derivative with *P*_*gap*_, *proB*, *T*_*pgk1*_, HygB^r^	This study
pδGAPg	pBluescript II KS(+) derivative with homologous δ region, *P*_*gap*_, *T*_*pgk1*_, G418^r^	Our lab
pδGAP′g	pBluescript II KS(+) derivative with homologous δ region, *P*_*gap*′_, *T*_*pgk1*_, G418^r^	Our lab
pδPGK1g	pBluescript II KS(+) derivative with homologous δ region, *P*_*pgk1*_, *T*_*pgk1*_, G418^r^	Our lab
pδGAL1g	pBluescript II KS(+) derivative with homologous δ region, *P*_*gal1*_, *T*_*pgk1*_, G418^r^	Our lab
pδGAPh	pBluescript II KS(+) derivative with homologous δ region, *P*_*gap*_, *T*_*pgk1*_, HygB^r^	Our lab
pδGAPg-gshF	pδGAPg derivative with *A. pleuropneumoniae gshF*, G418^r^	This study
pδGAP′g-gshF	pδGAP′g derivative with *A. pleuropneumoniae gshF*, G418^r^	This study
pδPGK1g-gshF	pδPGK1 g derivative with *A. pleuropneumoniae gshF*, G418^r^	This study
pδGAL1g-gshF	pδGAL1 g derivative with *A. pleuropneumoniae gshF*, G418^r^	This study
pδGAPh-gsh2gsh1	pδGAPh derivative with *GSH2*-*GSH1* from *S. cerevisiae*, HygB^r^	This study
pδGAPg-pro1gshB	pδGAPg derivative with *PRO1*-*gshB* from *S. cerevisiae* and *E. coli*, respectively, G418^r^	This study
pδGAPg-proBgshB	pδGAPg derivative with *proB*-*gshB* from *E. coli*, G418^r^	This study

^a^W303-1b/F/GAP and W303-1b/F represent the same engineered strain in this study

**Fig. 2:**
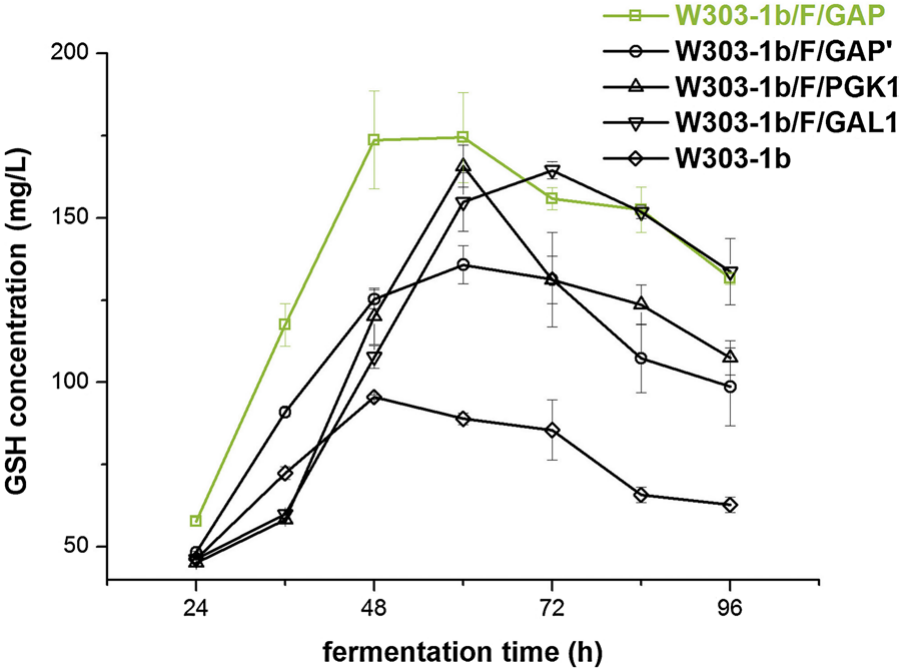
Evaluation of synthetic capacities of GSH of engineered *S. cerevisiae* strains harbouring *gshF* gene under the control of four different promoters. Intracellular GSH amount was monitored after 24-h fermentation and measured every 12 h. The values are presented as the means, and the *error bars* show the SD (n = 3)

GSH production in the engineered strains together with host strain W303-1b was compared as shown in Fig. 2. The four engineered strains conferred higher GSH concentrations than the parental strain. Among them, W303-1b/F/GAP (abbreviated to W303-1b/F in the following sections) produced higher intracellular GSH levels; the highest concentration was achieved after fermentation for 48–60 h. This result indicated that *A. pleuropneumoniae* GshF was active for GSH synthesis in *S. cerevisiae.* In addition, differences in synthetic capacities of GSH were considered to be directly related to the strengths of different promoters, thus GAP promoter of *S. cerevisiae* was utilized to keep uniform expression of each construct in this work.

### Active GK for GSH biosynthesis in *S. cerevisiae* W303-1b

In the cells lacking Gsh1-dependent biosynthetic pathway of GSH, it was found that γ-glutamyl phosphate is a common precursor shared by proline and GSH biosynthetic pathways. Thus, biosynthesis of proline could be partially diverted toward GSH production via γ-glutamyl phosphate (Fig. 1) and GK may play a central role in the alternative pathway.

**Fig. 3:**
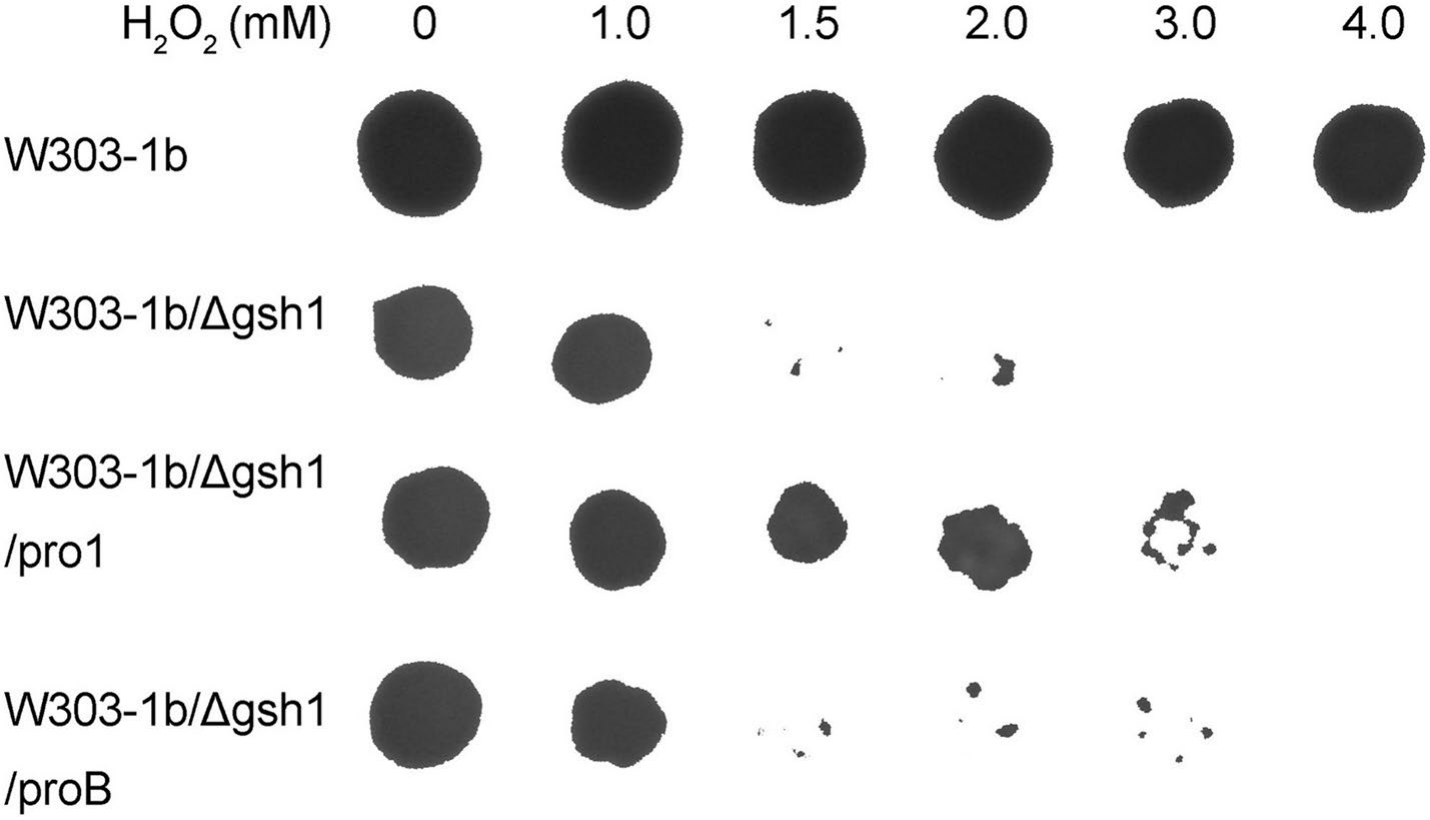
Patch assay on YPD containing the indicated amounts of H_2_O_2_. The tolerances to H_2_O_2_ of W303-1b (wt), W303-1b/Δgsh1 (*GSH1* deletion), W303-1b/Δgsh1/pro1 (*GSH1::P*_*gap*_-*PRO1*-*T*_*pgk1*_) and W303-1b/Δgsh1/proB (*GSH1::P*_*gap*_-*proB*-*T*_*pgk1*_) were evaluated

**Fig. 4:**
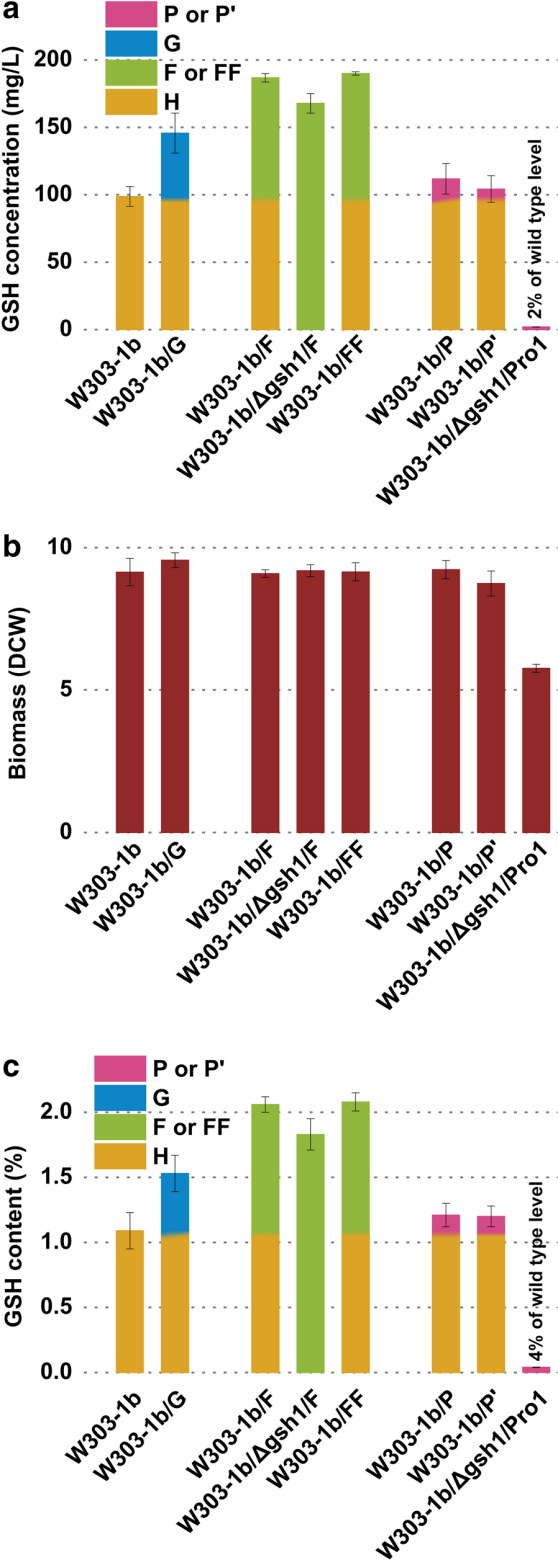
Single modulation of G pathway, F pathway and P pathway for GSH biosynthesis in *S. cerevisiae*. **a** GSH concentration (mg/L). **b** Biomass (DCW). **c** GSH content (%). *G* (*F* or *FF*, *P* or *P′*) represents improvement of GSH amount conferred by the overexpression of *G* (*F*, *P* or *P′*) pathway and H represents GSH amount of host strain W303-1b. DCW is calculated from OD_600_ values (OD_600_ = 10 corresponds to 1.19 g/L DCW). The values are presented as the means, and the *error bars* show the SD (n = 3)

Preliminary experiments had proved in vitro activity of GK for formation of intermediate γ-GC (data not shown). For the purpose of exploiting an active GK for biosynthesis of GSH in *S. cerevisiae* W303-1b, plasmids pΔgsh1h, pΔgsh1h-pro1 and pΔgsh1h-proB were constructed. With the homologous integration of the linearized plasmids, *GSH1* gene of wt W303-1b was deleted or replaced by recombinant DNA fragment of *S. cerevisiaePRO1* or *E. coli proB*, and the resulting strains were W303-1b/Δgsh1 (*GSH1* deletion), W303-1b/Δgsh1/pro1 (*GSH1::P*_*gap*_-*PRO1*-*T*_*pgk1*_) and W303-1b/Δgsh1/proB (*GSH1::P*_*gap*_-*proB*-*T*_*pgk1*_) (Table 2). Patch assay on YPD containing the given amounts of H_2_O_2_ was carried out with wt W303-1b, W303-1b/Δgsh1, W303-1b/Δgsh1/pro1 and W303-1b/Δgsh1/proB to detect their H_2_O_2_ tolerances that normally require GSH. As for W303-1b/Δgsh1/pro1 or W303-1b/Δgsh1/proB, the overexpression of introduced Pro1 or ProB under the control of strong constitutive GAP promoter might allow a small amount of GSH in the host strains conferring tolerance to oxidative stress. As shown in Fig. 3, all transformants grew on the plates with the concentration of H_2_O_2_ below 1.0 mM. However, their growth rates were apparently slower than wt strain (data not shown). W303-1b/Δgsh1 and W303-1b/Δgsh1/proB failed to grow when addition of H_2_O_2_ was more than 1.5 mM, while W303-1b/Δgsh1/pro1 with Pro1 overexpression construct afforded to form colonies on YPD containing 3.0 mM H_2_O_2_. The presence of GSH in W303-1b/Δgsh1/pro1 was about 4 % of wt level (Fig. 4c). This data showed that overexpression of Pro1 could produce more γ-glutamyl phosphate involved in an alternative pathway for a small amount of GSH formation in *S. cerevisiae* W303-1b.

### Overexpression of enzymes involved in GSH biosynthesis

In earlier studies, Gsh1 and Gsh2 (GshA and GshB) derived from yeast or *E. coli* were independently applied to various model microorganisms to generate genetically engineered strains with higher GSH productive capacities [15–19]. GshF from bacteria was also expressed in *E. coli* and yeast [20–22]. Here, *A. pleuropneumoniae* GshF was successfully expressed in *S. cerevisiae* as mentioned above. However, the recombinant constructs of Gsh2-Gsh1 and Pro1-GshB with a flexible linker have not been exploited for improving intracellular GSH synthesis until now.

To evaluate the potential of different pathways geared toward improving GSH synthesis, several δ DNA-mediated integrative expression vectors pδGAPh-gsh2gsh1, pδGAPg-pro1gshB or pδGAPg-proBgshB harbouring different pathway genes were constructed. Recombinant *S. cerevisiae* strains W303-1b/G, W303-1b/P or W303-1b/P′ (Table 2) were then generated by transforming different linearized plasmids into *S. cerevisiae* W303-1b as constructed W303-1b/F. The pathway genes with strong constitutive GAP promoters were introduced into the genome of W303-1b via homologous integration at δ-sites. To further compare GSH synthetic capacities of different pathways, *GSH1* gene of engineered strain W303-1b/F was deleted to eliminate synthesis of GSH by endogenous enzymes. The resulting strain W303-1b/Δgsh1/F (F pathway) together with W303-1b/G (G pathway) and W303-1b/Δgsh1/pro1 (P pathway) represent different pathway strains (Table 2). No dramatic differences in biomass between W303-1b/G, W303-1b/F, W303-1b/Δgsh1/F, W303-1b/P or W303-1b/P′ and original strain W303-1b were observed during shake flask cultures (Fig. 4). Three engineered strains W303-1b/G, W303-1b/F and W303-1b/P displayed higher activities for synthesizing GSH than the parental strain. The highest intracellular concentration of GSH was 186.77 mg/L produced by strain W303-1b/F, and most of the synthesized GSH could be attributed to the function of the introduced GshF as strain W303-1b/Δgsh1/F yielded a similar amount (about 90 %) GSH to W303-1b/F. Other strains W303-1b/G, W303-1b/P and W303-1b produced 145.72, 111.82 and 98.75 mg/L of GSH, respectively. Results of the synthetic capacities of different pathways (ranked in descending order) are as follows: F pathway, G pathway and P pathway.

Since W303-1b/F synthesized the highest GSH concentration among tested strains, it was chosen to examine the effect of gene dosage on GSH production. The antibiotic-resistant gene was removed from the genomic DNA of W303-1b/F using the Cre-*loxP* system, then W303-1b/F cells were further transformed with the linearized recombinant plasmid pδGAPg-gshF to obtain engineered strain W303-1b/FF (Table 2). Three recombinant strains harbouring two copies of *gshF* gene relative to that of W303-1b/F (Additional file 1: Table S8) were obtained and compared for their GSH production with W303-1b/F. However, no further improvement was observed (Fig. 4). This result suggests that increasing copy number was shown to be effective up to a certain limit of gene copy number.

### Three biosynthetic pathways combined to increase GSH production

To obtain a higher GSH concentration, three biosynthetic pathways of GSH were combinatorially expressed in *S. cerevisiae* W303-1b. Strain W303-1b/F with higher synthetic capacity of GSH was chosen as the starting strain for further introduction of other biosynthetic pathways. The two-pathway strains W303-1b/FG and W303-1b/FP were obtained by the integration of linearized pδGAPh-gsh2gsh1 and pδGAPg-pro1gshB, respectively. Further integration of linearized pδGAPg-pro1gshB into the engineered W303-1b/FG generated the three-pathway strain W303-1b/FGP (Table 2).

**Fig. 5:**
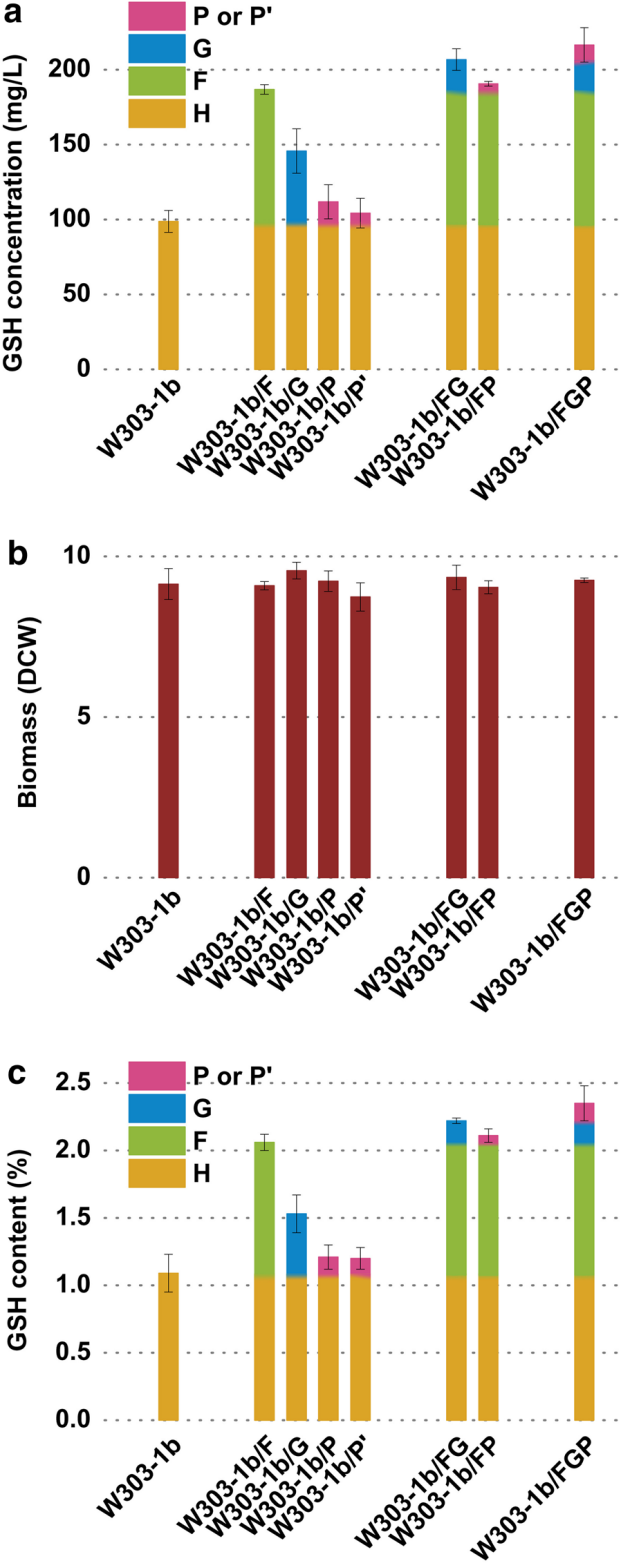
GSH production of single-pathway, two-pathway and three-pathway engineered strains. **a** GSH concentration (mg/L). **b** Biomass (DCW). **c** GSH content (%). *G* (*F*, *P* or *P′*) represents improvement of GSH amount conferred by the overexpression of *G* (*F*, *P* or *P′*) pathway and *H* represents GSH amount of host strain W303-1b. DCW is calculated from OD_600_ values (OD_600_ = 10 corresponds to 1.19 g/L DCW). The values are presented as the means, and the *error bars* show the SD (n = 3)

As expected, strains W303-1b/FG, W303-1b/FP and W303-1b/FGP yielded 206.70, 190.57 and 216.50 mg/L of GSH in shake flask cultures, indicating that introduction of a second pathway could enhance the synthetic capacity of GSH. Among all the engineered strains, the strain W303-1b/FGP with three biosynthetic pathways showed the highest GSH producing capacity and improved the intracellular GSH concentration by 2.19-fold compared with W303-1b (Table 3; Fig. 5).

**Table 3:** Biomass, GSH concentration, GSH content and molar ratio of γ-GC to GSH of various strains in flask cultures with or without addition of amino acid precursors (AA)

Strains	Biomass (DCW)^a^	GSH concentration (mg/L)	GSH content (%)	Molar ratio of γ-GC to GSH (%)
W303-1b	9.14 ± 0.48	98.75 ± 7.30	1.09 ± 0.14	6.74 ± 0.24
W303-1b/F	9.09 ± 0.13	186.77 ± 3.19	2.05 ± 0.06	10.29 ± 0.87
W303-1b/G	9.56 ± 0.26	145.72 ± 14.87	1.52 ± 0.14	9.72 ± 0.93
W303-1b/P	9.23 ± 0.32	111.82 ± 11.34	1.21 ± 0.09	8.58 ± 2.53
W303-1b/P′	8.74 ± 0.44	104.24 ± 9.86	1.19 ± 0.08	7.34 ± 0.42
W303-1b/FG	9.35 ± 0.38	206.70 ± 7.23	2.21 ± 0.02	11.26 ± 0.67
W303-1b/FP	9.04 ± 0.20	190.57 ± 1.56	2.11 ± 0.05	10.92 ± 0.93
W303-1b/FGP	9.26 ± 0.07	216.50 ± 11.46	2.34 ± 0.13	9.97 ± 0.40
W303-1b/F (AA)	8.58 ± 0.09	252.33 ± 28.62	2.94 ± 0.36	9.65 ± 0.51
W303-1b/FGP (AA)	8.38 ± 0.37	316.77 ± 45.69	3.77 ± 0.43	9.50 ± 1.43

^a^DCW is calculated from OD_600_ values (OD_600_ = 10 corresponds to 1.19 g/L DCW). The values given are mean values ± standard deviation

### Shake flask culture with addition of amino acid precursors

**Fig. 6:**
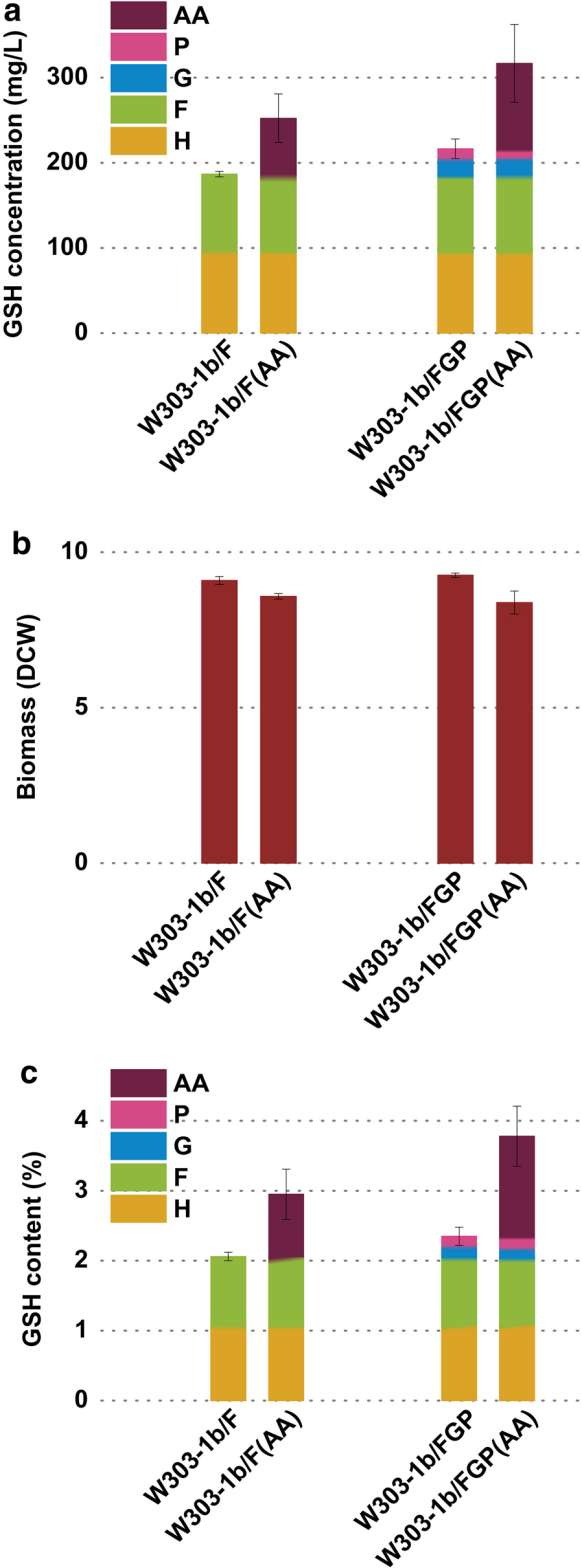
Evaluation of synthetic capacities of GSH of engineered *S. cerevisiae* W303-1b/F and W303-1b/FGP in shake flask cultures with or without addition of three amino acid precursors. **a** GSH concentration (mg/L). **b** Biomass (DCW). **c** GSH content (%). *G* (*F* or *P*) represents improvement of GSH content conferred by overexpression of *G* (*F* or *P*) pathway, *H* represents GSH content of host strain W303-1b and *AA* indicates improvement of GSH amount conferred by addition of amino acid precursors. DCW is calculated from OD_600_ values (OD_600_ = 10 corresponds to 1.19 g/L DCW). The values are presented as the means, and the *error bars* show the SD (n = 3)

Genetic manipulation and medium optimization together with the addition of amino acid precursors are required to increase GSH production. In order to further investigate the potential of three-pathway combinatorial biosynthesis of GSH, we compared utilization and conversion efficiency of amino acid precursors of engineered strain W303-1b/FGP with that of strain W303-1b/F in shake flask cultures. Figure 6 showed that the biomass of both W303-1b/FGP and W303-1b/F dropped by around 10 % when three amino acid precursors were added, and GSH contents of W303-1b/FGP and W303-1b/F were 3.77 and 2.94 %, respectively. After addition of amino acid precursors, GSH production of W303-1b/FGP was improved by 61.37 %, while that of W303-1b/F was 43.17 %. This result indicated that the engineered strain W303-1b/FGP held a higher utilization and conversion efficiency of amino acid precursors. That is to say, advantages of W303-1b/FGP over W303-1b/F include higher GSH yield and utilization and conversion efficiency of amino acid precursors.

### Batch culture of engineered strain with three biosynthetic pathways

**Fig. 7:**
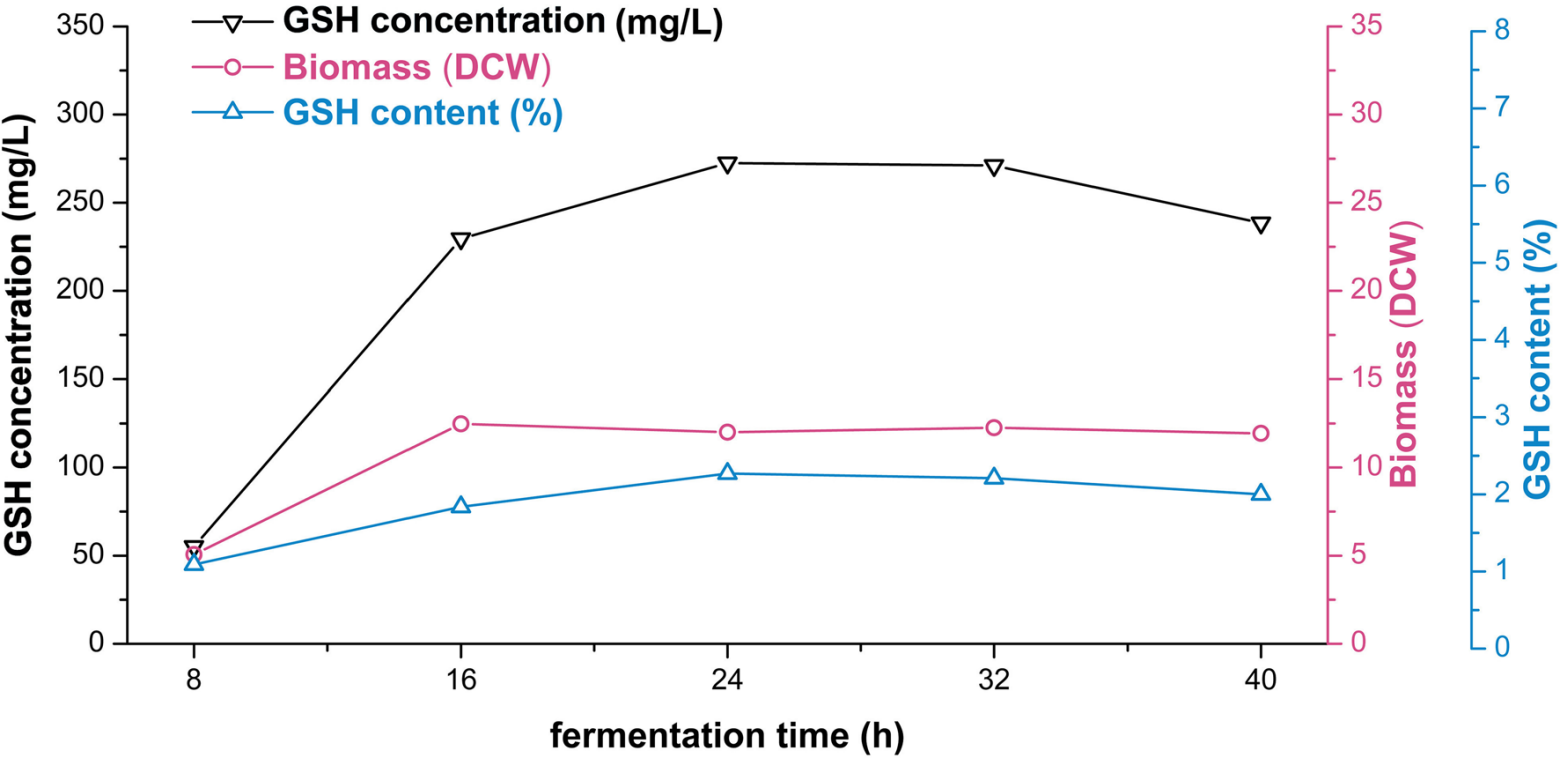
Batch culture of *S. cerevisiae* W303-1b/FGP in 10-L fermentor. The working volume was 4 L. Culture temperature and DO were controlled at 30 °C and 25 %, and pH was uncontrolled. Intracellular GSH amount was measured every 8 h

To examine the GSH productive capacity of engineered strain W303-1b/FGP in a larger scale, batch cultivation for GSH production was carried out in a 10-L fermentor. The time course of batch culture with W303-1b/FGP was shown in Fig. 7. Both GSH concentration and biomass were higher than that of shake flask culture. The GSH concentration increased to the highest point of 272.52 mg/L after fermentation for 24 h, and the GSH content was 2.27 %. It was observed that the GSH productive capacity measured by GSH content showed no obvious change after scale-up fermentation.

## Discussion

Recently, a novel bifunctional enzyme GshF has aroused concerns about GSH productive capacity as most reported GshFs are insensitive to feedback inhibition of GSH except that of *P. multocida* and *L. monocytogenes* [8–11, 20]. In this study, the putative *gshF* from *A. pleuropneumoniae* resynthesized according to codon usage of *S. cerevisiae* was firstly functionally expressed in *S. cerevisiae*, and then the relationship between GSH production and the strengths of three different glucose-based promoters, including GAP and PGK1 promoters of *S. cerevisiae* and GAP promoter from *P. pastoris* (GAP′), and a galactose-based GAL1 promoter of *S. cerevisiae* was analyzed. Figure 2 showed that the expressed GshF under the control of constitutive GAP promoter of *S. cerevisiae* produced the highest concentration of GSH. Thus it is presumed that the expression of GshF driven by GAP promoter of *S. cerevisiae* functioned well for GSH synthesis. Through the comparison of the productive capacity of GSH of engineered strain W303-1b/F with that of the reported GS115/LmgshF with *L. monocytogenes* GshF, an observed variance was that F pathway presented higher synthetic capacity of GSH than G pathway (Fig. 4) [21]. This conflicting result may be attributed to the different sensitivities of GshFs from *L. monocytogenes* and *A. pleuropneumoniae* to feedback inhibition of GSH [8, 11]. Since single F pathway could produce higher amounts of GSH compared with other pathways, we tried to augment the copies of F pathway by further integration of *gshF* to improve the productive capacity of GSH of engineered strain W303-1b/F. However, the *gshF* copy number increase didn’t further improve GSH concentration in W303-1b/F, indicating that a reasonable rather than high copy number of *gshF* gene driven by GAP promoter is sufficient for the highest expression levels and synthetic capacity of GSH through one pathway is limited.

It has been reported that proline biosynthesis could contribute to GSH synthesis. γ-glutamyl phosphate is required as the common intermediate for the crosstalk between proline biosynthetic pathway and conventional Gsh1/Gsh2 pathway. Veeravalli et al. reported that, different from in vitro assay, as l-cysteine is present in micromolar concentrations in the intracellular environment, the activity of γ-glutamyl phosphate reductase (GPR) encoded by *PRO2* in eukaryotes or *proA* in prokaryotes must be shut off to allow γ-GC formation from γ-glutamyl phosphate [12]. In this study, as amino acid precursors could be fed to improve concentrations of intracellular free l-cysteine, we resorted to overexpression of GK instead of loss-of-function mutations in GPR for accumulation of γ-glutamyl phosphate to strengthen the redirection of γ-glutamyl phosphate to GSH synthesis. It was indirectly confirmed by the result of patch assay that W303-1b/Δgsh1/pro1 with Pro1 overexpression construct performed a relatively stronger H_2_O_2_ tolerance than W303-1b/Δgsh1/proB and W303-1b/Δgsh1 (Fig. 3). Overexpressed Pro1 mediated accumulation of γ-glutamyl phosphate which was partially diverted toward GSH production, while heterologous ProB (optimized according to codon usage of *S. cerevisiae*) did not work well. Consistent with the result of patch assay, W303-1b/P provided a slightly higher GSH production than W303-1b/P′ and strain W303-1b in shake flask cultures (Fig. 4). When P pathway was upregulated, the accumulated γ-glutamyl phosphate could improve utilization of extra l-cysteine by addition of amino acid precursors for improvement of GSH synthesis. Thus, P pathway could be modulated for improving intracellular GSH synthesis.

Previous studies have reported individual modulation of G pathway and F pathway for GSH production [15–22]. In this study, P pathway was exploited for GSH production for the first time. Combining G, F and P three pathways is a novel method for GSH production. Genes of three pathways were uniformly regulated and expressed by exactly the same strategy to evaluate synthetic capacities of engineered strains with different combinations of biosynthetic pathways of GSH. As expected, the engineered strain W303-1b/FGP presented the highest yield of GSH. Moreover, the result of shake flask cultures with addition of amino acid precursors (Fig. 6) further proved the advantages of W303-1b/FGP. In batch culture process, the recombinant strain W303-1b/FGP also afforded high efficiency in GSH production and reached an intracellular GSH content of 2.27 % after 24-h fermentation. Generally, a GSH content of 3–5 % could be obtained using strains selected via classical selection strategy, but the exact mechanisms underlying higher GSH accumulation in these mutants has remained rather obscure. Nisamedtinov et al. reported that GSH over-accumulation in *S. cerevisiae* mutants selected via random mutagenesis is caused by the combined effect of higher cysteine synthesis and increased flux through Gsh1 reaction [25]. These genetic manipulations could confer a GSH content of 1–2 % on the engineered strains. The GSH synthetic capacity of engineered strain W303-1b/FGP constructed in this study was comparable to that of reported genetically engineered strains and higher than that of engineered strains with single modulation of G pathway or F pathway (Table 1). Increase in GSH content of W303-1b/FGP may be attributed to the fact that the three pathways form a complex network of GSH biosynthesis producing comprehensive effects. Here, it should be noted that the balance of two consecutive enzymatic reactions especially in the complex network of GSH biosynthesis is very important. Previous studies have reported that overexpression of conventional Gsh1/Gsh2 pathway improved production of intracellular GSH along with accumulation of intermediate γ-GC which increased significantly when amino acid precursors were added [21]. In our study, as the intracellular γ-GC level was only increased slightly compared with that of original strain W303-1b when F pathway was introduced, we artificially introduced a flexible six-glycine linker between the two enzymes of G pathway as well as P pathway to improve the coupling efficiency by mimicking the native domain fusion of GshF. As expected, intracellular γ-GC amounts of W303-1b/FGP were still maintained at low levels even when amino acid precursors were added (Table 3).

## Conclusions

In summary, a three-pathway combinatorial method of GSH biosynthesis presents higher GSH yield and utilization efficiency of amino acid precursors than that of a single pathway. This strategy is an important development toward improving industrial production of GSH using *S. cerevisiae*.

## Methods

### Strains

*E. coli* Trans1-T1 (TransGen, Beijing, China) was used for propagation and manipulation of the recombinant DNA. *S. cerevisiae* W303-1b (*MATα ade2*-*1 leu2*-*3*,*112 his3*-*11*,*15 ura3*-*1 trp1*-*1*) was used as host strain for DNA transformation and expression of recombinant genes. Yeast transformants were grown at 30 °C in YPD medium (10 g/L yeast extract, 20 g/L tryptone and 20 g/L glucose).

### Plasmid construction and yeast transformation

All plasmids were constructed using conventional restriction enzyme-mediated cloning methods. Based on the nucleotide sequences of the target genes, development of primer sets were designed and used to amplify gene fragments by PCR (Additional file 1: Tables S1–S6). δ DNA-mediated integrative expression vectors as described in Ref. [26], and plasmids for deletion of *GSH1* gene were constructed as shown in Additional file 1: Figures S1–S4. The resulting plasmids were linearized by digestion with restriction enzyme *Not*I and transformed into *S. cerevisiae* using lithium acetate method. The positive transformants were selected on YPD plates with addition of antibiotics geneticin (G418, 4 mg/mL) or hygromycin B (HygB, 1 mg/mL) and verified by DNA sequencing after the integrated gene fragments were amplified by PCR using the corresponding primers and the genomic DNA templates. The copy numbers of integrated genes were verified using real-time PCR (primers GshF-RT1/GshF-RT2, Additional file 1: Table S7) and calculated by a comparative *C*_T_ method [27]. For each transformation, three positive colonies were picked randomly for further investigation. The repeated use of the marker genes were performed via a loxP-marker-loxP gene disruption cassette according to Ref. [28].

### Patch assay

Single colonies of strains W303-1b, W303-1b/Δgsh1, W303-1b/Δgsh1/pro1 and W303-1b/Δgsh1/proB were diluted in distilled H_2_O and patched (about 500 cells/μL) on YPD plates containing various concentrations of H_2_O_2_. Plates were incubated for about 72 h at 30 °C to compare H_2_O_2_ tolerances by watching their growth.

### GSH production of the engineered *S. cerevisiae* strains in shake flasks

Three positive colonies of each engineered *S. cerevisiae* strain were cultured at 30 °C in shake flasks containing 20 mL of liquid YPD medium with agitation at 250 rpm for 18–24 h for primary culture. An adequate volume of fresh cultures were then inoculated into a set of 250-mL flasks containing 50 mL of liquid YPD medium to keep an initial optical density at 600 nm (OD_600_) value of 0.2. The cultures were incubated at 30 °C with agitation at 250 rpm for additional 48 h. In order to further increase the production of GSH, the addition of amino acid precursors (5 mM glutamic acid, 5 mM cysteine and 5 mM glycine) was single shot after 36-h cultivation for feeding assay.

### Batch culture of the engineered strain W303-1b/FGP for GSH production

The batch fermentation was operated in a 10-L fermentor (China Beauty). Liquid YPD medium for batch culture was sterilized by autoclave at 121 °C for 20 min. Engineered strain W303-1b/FGP grown on YPD agar plate was inoculated into liquid YPD medium and cultured at 30 °C with agitation at 250 rpm for 18–24 h. 10 % (v/v) seed cultures were transferred into the fermentor. The dissolved oxygen (DO) was maintained at 25 % through adjustment of stirrer speed (100–500 rpm) and the aeration rate (1–5 L/h). The temperature was set to 30 °C and the pH was uncontrolled.

### Analytical methods

Grown cultures were removed from the incubator at a given time and biomass was measured as optical density at 600 nm (OD_600_) and/or the dry cell weight (DCW). OD_600_ was determined after the sample was appropriately diluted. DCW (g/L) was determined after yeast cells were centrifuged at 4000 rpm for 10 min and washed twice with distilled water before drying at 105 °C to constant weight. OD_600_ was converted to DCW using calibration coefficient (OD_600_ = 10, equivalent to 1.19 g/L DCW in this study). To monitor the intracellular GSH and γ-GC amounts, cells were harvested and washed with distilled water. Intracellular GSH and γ-GC were extracted from the cells by 40 % ethanol for 2 h at 30 °C. The GSH and γ-GC concentrations (mg/L) of the sample were measured using the 4-fluoro-7-aminosulfonylbenzofurazan (ABD-F) derivatization method (Additional file 1: Figure S5) according to a previous report [29] with the reduced GSH as the standard. The GSH content (%) was calculated from the intracellular GSH concentration (mg/L) divided by DCW.

## Additional file

**Additional file 1.** **Table S1.** Primers used for cloning of *A. pleuropneumoniae gshF* gene optimized according to codon usage of *S. cerevisiae.* **Table S2.** Primers used for cloning of *S. cerevisiae GSH2* gene. **Table S3.** Primers used for cloning of *S. cerevisiae GSH1* gene. **Table S4.** Primers used for cloning of *S. cerevisiae PRO1* gene. **Table S5.** Primers used for cloning of *E. coli proB* mutant optimized according to codon usage of *S. cerevisiae.* **Table S6.** Primers used for cloning of *E. coli gshB* mutant optimized according to codon usage of *S. cerevisiae.* **Table S7.** Primers used for real-time PCR to verify copy number of *gshF* gene. **Table S8.** Relative quantitation of copy number of *gshF* gene. **Figure S1.** Construction of integrative expression vectors pδGAPg-gshF, pδGAP′g-gshF, pδGAL1g-gshF and pδPGK1g-gshF used to evaluate effects of different promoter strengths on the activity of GshF. **Figure S2.** Construction of integrative expression vector pδGAPh-gsh2gsh1 for overexpression of Gsh2-Gsh1 fusion protein. **Figure S3.** Construction of integrative expression vectors pδGAPg-pro1gshB and pδGAPg-proBgshB for overexpression of Pro1-GshB and ProB-GshB fusion proteins. **Figure S4.** Construction of plasmids pΔgsh1h-pro1, pΔgsh1h-proB and pΔgsh1h used for deletion of *GSH1* gene. **Figure S5.** Chromatograms of standard thiols and samples labelled with ABD-F.
