# Supplementary material for: Three-pathway combination for glutathione biosynthesis in Saccharomyces cerevisiae
Source: Microb Cell Fact. 2015 Sep 16;14:139. doi: 10.1186/s12934-015-0327-0 (PMC4574134; doi:10.1186/s12934-015-0327-0)
Supplement: Supplementary file 1 — Additional file 1. Table S1. Primers used for cloning of A. pleuropneumoniae gshF gene optimized according to codon usage of S. cerevisiae. Table S2. Primers used for cloning of S. cerevisiae GSH2 gene. Table S3. Primers used for cloning of S. cerevisiae GSH1 gene. Table S4. Primers used for cloning of S. cerevisiae PRO1 gene. Table S5. Primers used for cloning of E. coli proB mutant optimized according to codon usage of S. cerevisiae. Table S6. Primers used for cloning of E. coli gshB mutant optimized according to codon usage of S. cerevisiae. Table S7. Primers used for real-time PCR to verify copy number of gshF gene. Table S8. Relative quantitation of copy number of gshF gene. Figure S1. Construction of integrative expression vectors pδGAPg-gshF, pδGAP′g-gshF, pδGAL1g-gshF and pδPGK1g-gshF used to evaluate effects of different promoter strengths on the activity of GshF. Figure S2. Construction of integrative expression vector pδGAPh-gsh2gsh1 for overexpression of Gsh2-Gsh1 fusion protein. Figure S3. Construction of integrative expression vectors pδGAPg-pro1gshB and pδGAPg-proBgshB for overexpression of Pro1-GshB and ProB-GshB fusion proteins. Figure S4. Construction of plasmids pΔgsh1h-pro1, pΔgsh1h-proB and pΔgsh1h used for deletion of GSH1 gene. Figure S5. Chromatograms of standard thiols and samples labelled with ABD-F. [file 12934_2015_327_MOESM1_ESM.pdf]

ADDITIONAL FILE 1

List of tables

Table S1. Primers used for cloning of *A. pleuropneumoniae gshF* gene optimized according to codon usage of *S. cerevisiae.*

Table S2. Primers used for cloning of *S. cerevisiae GSH2* gene.

Table S3. Primers used for cloning of *S. cerevisiae GSH1* gene.

Table S4. Primers used for cloning of *S. cerevisiae PRO1* gene.

Table S5. Primers used for cloning of *E. coli proB* mutant optimized according to codon usage of *S. cerevisiae.*

Table S6. Primers used for cloning of *E. coli gshB* mutant optimized according to codon usage of *S. cerevisiae.*

Table S7. Primers used for real-time PCR to verify copy number of *gshF* gene.

Table S8. Relative quantitation of copy number of *gshF* gene.

List of figures

Figure S1. Construction of integrative expression vectors pδGAPg-gshF, pδGAP'g-gshF, pδGAL1g-gshF and pδPGK1g-gshF used to evaluate effects of different promoter strengths on the activity of GshF.

Figure S2. Construction of integrative expression vector pδGAPh-gsh2gsh1 for overexpression of Gsh2-Gsh1 fusion protein.

Figure S3. Construction of integrative expression vectors pδGAPg-pro1gshB and pδGAPg-proBgshB for overexpression of Pro1-GshB and ProB-GshB fusion proteins.

Figure S4. Construction of plasmids pΔgsh1h-pro1, pΔgsh1h-proB and pΔgsh1h used for deletion of *GSH1* gene.

Figure S5. Chromatograms of standard thiols and samples labelled with ABD-F.

Table S1. Primers used for cloning of *A. pleuropneumoniae gshF* gene optimized according to codon usage of *S. cerevisiae.*

Primers	Oligonucleotide sequences (5'-3')
APF1_1	GAATTCCATATGAAATTACAACAGTTGATTAAAACTCATCACTTAGGTTTGCTATT
APF1_2	CATCACTTAGGTTTGCTATTTCAACAGGGTAAATTTGGCATCGAAAAGGAATCTCAAAG
APF1_3	CGAAAAGGAATCTCAAAGAATTGATAATAAAGGTAATATTGTTACTACAGCCCATCCTT
APF1_4	TTACTACAGCCCATCCTTCTGTTTTTGGTAACAGATCATATCATCCATATATTCAGACC
APF1_5	CATCCATATATTCAGACCGATTTTGCAGAAAGTCAGTTAGAATTAATTACACCACCTAA
APF1_6	ATTAATTACACCACCTAACGATACATTGGAAGACACATATAGATGGCTATCTGCTATTC
APF1_7	GATGGCTATCTGCTATTCATGAGGTAACATTAAGATCATTGCCAGATGATGAATATATT
APF1_8	CCAGATGATGAATATATTTTCCCATTTTCTATGCCTGCCGGTTTACCGCCAGAATTTGA
APF1_9	TTTACCGCCAGAATTTGAAATTAAAGAGGCACAATTAGATAACGAATGGGACGTGAAAT
APF1_10	TTGTATTTGCCATAAATGGCAGACAAATGTTCTCTATATTTCACGTCCCATTCGTTATCGATAACGAATGGGACGTGAAATATAGAGAACATTTGTCTGCCATTTATGGCAAATACAA
APF1_11	CAGAAATTTGGAAATTATAGTGAATACCTGACACCATTTGCTTGTATTTGCCATAAATGCATTTATGGCAAATACAAGCAAATGGTGTCAGGTATTCACTATAATTTCCAAATTTCTG
APF1_12	GTATTCCGTTTGTAATGCAAATGTAGATTCGACAAACTCTTCAGAAATTTGGAAATTATATAATTTCCAAATTTCTGAAGAGTTTGTCGAATCTACATTTGCATTACAAACGGAATAC
APF1_13	AATTCCATATATAACGCATTTCTGAACGCAATTTTATCTCTGTATTCCGTTTGTAATGCGCATTACAAACGGAATACAGAGATAAAATTGCGTTCAGAAATGCGTTATATATGGAATT
APF1_14	AATAAACTAAAATCCATTGGTATCTTAAAAAGTTATTGGCTAATTCCATATATAACGCATGCGTTATATATGGAATTAGCCAATAACTTTTTAAGATACCAATGGATTTTAGTTTATT
APF1_15	ACCGAAATATTGCGCTTCTACAGTTGGGGTTGCGGCTAACAAATAAACTAAAATCCATTAATGGATTTTAGTTTATTTGTTAGCCGCAACCCCAACTGTAGAAGCGCAATATTTCGGT
APF1_16	AAACTTCTTACTAATTGACCTTCTGCCAATGGTGAGTTTTTACCGAAATATTGCGCTTCGAAGCGCAATATTTCGGTAAAAACTCACCATTGGCAGAAGGTCAATTAGTAAGAA

	GTTT
APF1_17	CAATATGTGGCGCATTTACATAACCATAAGGGCCAGACCTTAAACTTCTTACTAATTGATCAATTAGTAAGAAGTTTAAGGTCTGGCCCTTATGGTTATGTAAATGCGCCACATATTG
APF1_18	AAAGAGGTCGACATATTGTTGCAAGCTGTCGTGGTTGATCACAATATGTGGCGCATTTATAAATGCGCCACATATTGTGATCAACCACGACAGCTTGCAACAATATGTCGACCTCTTT
APF2_1	GAATTCCTCGAGTCTTTAGAACATTTTGTAGCAACCGGCGAT
APF2_2	AACATTTTGTAGCAACCGGCGATTTGTTGGCAGAAAAAGAATTTTATTCAAACGTTAGA
APF2_3	TTTTATTCAAACGTTAGATTAAGGGGTGCGAAAAAGGCAAGAAAATTGTTAGAGAAGGG
APF2_4	AAAATTGTTAGAGAAGGGTGTTAAATATGCGGAATTTAGATTATTTGATTTGAATCCTT
APF2_5	TATTTGATTTGAATCCTTTTTCTCCTTACGGTATCGAATTAGCAGACGCGAAATTTATT
APF2_6	GCAGACGCGAAATTTATTCATTTGTTCTTATTGGCGATGTTGTGGATGGATGAAACATC
APF2_7	GTGGATGGATGAAACATCTGGTCAAAGAGAAGTCGAAATTGGTACACAAAAATTATACC
APF2_8	GTACACAAAAATTATACCAAGTTGCCTTGGAAGATCCTAGATCACACACTGCGTTCCAA
APF2_9	TCACACACTGCGTTCCAAGCAGAGGGTGAGGCGATCTTGAACTTAATGTTGGCAATGTT
APF2_10	CTTAATGTTGGCAATGTTAGACGATTTGTCTGTACCACAAAACGAGAAAGATTTATTGC
APF2_11	ACGAGAAAGATTTATTGCAACAAAAATTGGCACAATTTGCCGATCCTTCACAAACTGTA
APF2_12	TAAGAGCCGGCTTGTTCGACTGCGGCCAATAATCTACCGTTTACAGTTTGTGAAGGATCGATCCTTCACAAACTGTAAACGGTAGATTATTGGCCGCAGTCGAACAAGCCGGCTCTTA
APF2_13	GCGCTTTATATTGTTGAGCTAATTGTGCACCCAATGCTTTATAAGAGCCGGCTTGTTCGCGAACAAGCCGGCTCTTATAAAGCATTGGGTGCACAATTAGCTCAACAATATAAAGCGC
APF2_14	ATTATCGAAAGCAGAAATCGCATAAAATCTCTCGAATGCTTGCGCTTTATATTGTTGAGCTCAACAATATAAAGCGCAAGCATTCGAGAGATTTTATGCGATTTCTGCTTTCGATAAT
APF2_15	TGGATCGCATCAAATAACAAAGCCTGTGTAGACAACTCCATATTATCGAAAGCAGAAATATTTCTGCTTTCGATAATATGGAGTTGTCTACACAGGCTTTGTTATTTGATGCGATCCA
APF2_16	ACTGATCGTTTTCATCTAACAATTCGATCTGTAAGCCTTGTTGGATCGCATCAAATAACGTTATTTGATGCGATCCA

	ACAAGGCTTACAGATCGAATTGTTAGATGAAAACGATCAGT
APF2_17	TTTCACATATTCCAAATGATCGCCAAATTTTAATGCCAAGAACTGATCGTTTTCATCTATAGATGAAAACGATCAGTTCTTGGCATTAAAATTTGGCGATCATTTGGAATATGTGAAA
APF2_18	AATGGCGAAATATACTGATCGTGAGAGGTCATATTGCCGTTTTTCACATATTCCAAATGCATTTGGAATATGTGAAAAACGGCAATATGACCTCTCACGATCAGTATATTTCGCCATT
APF2_19	TCGCCAACACCTTTTTGGTTACGACTTTGTTTTCCATAATTAATGGCGAAATATACTGATCAGTATATTTCGCCATTAATTATGGAAAACAAAGTCGTAACCAAAAAGGTGTTGGCGA
APF2_20	AGAGGTAAATTCAACAGATTTTGGCACATTAAAACCGGCTTTCGCCAACACCTTTTTGGCCAAAAAGGTGTTGGCGAAAGCCGGTTTTAATGTGCCAAAATCTGTTGAATTTACCTCT
APF2_21	TTACCTTCAAATAATGGATAGTGTGCCACCGCTTGTTCTACAGAGGTAAATTCAACAGATCTGTTGAATTTACCTCTGTAGAACAAGCGGTGGCACACTATCCATTATTTGAAGGTAA
APF2_22	ATCATGGTTAACCACCGCTTTACCTTCAAATAATGGATAGT
APF3_1	GATATAAATATTAAGCCTAAATCAACTAATTACGGCTTAGGT
APF3_2	AAATCAACTAATTACGGCTTAGGTATTACAATTTTCCAGCAAGGCGTGACGGATAAAGC
APF3_3	AGGCGTGACGGATAAAGCCGACTTTGCCAAAGCGATTGAAATTGCGTTCAGAGAAGATA
APF3_4	TTGCGTTCAGAGAAGATAAAGAAGTGATGGTGGAAGACTATTTAGTCGGCACCGAATAC
APF3_5	TTAGTCGGCACCGAATACAGATTCTTTGTGTTAGGCGATGAAACATTGGCGGTATTGTT
APF3_6	AACATTGGCGGTATTGTTAAGAGTGCCAGCAAATGTGAAAGGTGATTGTATACATACAG
APF3_7	GTGATTGTATACATACAGTGAGAGAATTGGTGGAAGCGAAAAACTCAGATCCATTGAGA
APF3_8	AACTCAGATCCATTGAGAGGTGACGGCTCAAGATCACCATTGAAGAAAATCGCCTTAGG
APF3_9	GAAGAAAATCGCCTTAGGTGATATTGAATTGTTACAATTGAAAGAGCAAGGTTTAACGC
APF3_10	TCTTAATTGTACGATTTGACCATCAGCAGGAATAGAATCAGGCGTTAAACCTTGCTCTTAAGAGCAAGGTTTAACGCCTGATTCTATTCCTGCTGATGGTCAAATCGTACAATTAAGA
APF3_11	ATATCAATTGAATCACCGCCGGTAGAAATATTAGAGTTGGCTCTTAATTGTACGATTTGCAAATCGTACAATTAAGAGCCAACTCTAACATTTCTACCGGCGGTGATTCAATTGATAT

APF3_12	CGACCGCTAATTGTTTATAACTGTCATGCATTTGATCAGTCATATCAATTGAATCACCGCGGTGATTCAATTGATATGACTGATCAAATGCATGACAGTTATAAACAATTAGCGGTCG
APF3_13	ATCCACACCGCAGACTTTTGCACCCATTTCTTTGGCAATACCGACCGCTAATTGTTTATATAAACAATTAGCGGTCGGTATTGCCAAAGAAATGGGTGCAAAAGTCTGCGGTGTGGAT
APF3_14	AAAGAAGGTTCAGCGGCTTTGGTTAAATCTGGAATGATTAAATCCACACCGCAGACTTTAAAGTCTGCGGTGTGGATTTAATCATTCCAGATTTAACCAAAGCCGCTGAACCTTCTTT
APF3_15	TCATAGGATTAAAGTTTGCTTCAATCACACCCCATGATCTCAAAGAAGGTTCAGCGGCTAGCCGCTGAACCTTCTTTGAGATCATGGGGTGTGATTGAAGCAAACTTTAATCCTATGA
APF3_16	CCTTCTTGATTTTCCTTGGTAAGGGAAAATATGCATCATCATCATAGGATTAAAGTTTGCAAACTTTAATCCTATGATGATGATGCATATTTTCCCTTACCAAGGAAAATCAAGAAGG
APF3_17	GGCAATTCTGGAAACAACATTTTTAACACGGCTTTGGTCAACCTTCTTGATTTTCCTTGCAAGGAAAATCAAGAAGGTTGACCAAAGCCGTGTTAAAAATGTTGTTTCCAGAATTGCC
APF3_18	GGATCCGGCTAGCTTAAGGCAATTCTGGAAACAACATTTT

Table S2. Primers used for cloning of *S. cerevisiae GSH2* gene.

Primers	Oligonucleotide sequences (5'-3')
GSH2_1	GATCCTCCAAAGGTAGCAAAGTG
GSH2_2	AAGTTCTAGCATCATCTTCCTAGC
GSH2_3	AAGGATATCATATGGCACACTATCCACCTTCC
GSH2_4	AGGATCACTAGTTGTAGAAGAACTT
GSH2_5	TCTACGGCTAGCGATCCTATTGTCGCATTCATT
GSH2_6	CCTATTGTCGCATTCATTGTGCAAAGAAACGAGAGAAATGTGTTTGATCAAAAGGTCTT
GSH2_7	GTTTGATCAAAAGGTCTTGGAATTGAATTTATTGGAAAAATTCGGTACTAAATCTGTTA
GSH2_8	TCGGTACTAAATCTGTTAGGTTGACTTTTGATGATGTTAATGATAAATTGTTTATTGAT
GSH2_9	TCCTGCTCTGTGTCCCTAATGAATAATTTTCCAGTTTTATCATCAATAAACAATTTATC
GSH2_10	CAGTGGTTGTGTAACCCGTTCTGTAATAAACCACCGCTATTTCCTGCTCTGTGTCCCTA
GSH2_11	CAAGAATAGTCTTGCCTCCCAGTCCTTTTCAGAAGTGTAATCAGTGGTTGTGTAACCCG
GSH2_12	TGCGAAACTTTTTTCCAAGAATAGTCTTGCCTCC
GSH2_13	TCTTGGAAAAAAGTTTCGCAATAAAGGCCCCAGATTTATTGACTCAATTATCTGGCTCC
GSH2_14	CTCTTGCCTTCCCTGCCAAGTTTCGTATCATCCAAGGGATA
GSH2_15	ACTTGGCAGGGAAGGCAAGAG
GSH2_16[a]	TACACGGGATCCGCCACCTCCACCTCCGTAAAGAATAATACTGTCCAAA

[a] Nucleotide sequence encoding the linker is underlined.

Table S3. Primers used for cloning of *S. cerevisiae GSH1* gene.

Primers	Oligonucleotide sequences (5'-3')
GSH1_1	GAGCAGATTTAGTATAGGGCTAC
GSH1_2	ATTCCAGGATCCATGGGATTGTTAGCTTTGGGCACGCCTTTGC
GSH1_3	CTTGTCATGGCAAACGTCCAACATAGAATTTCTCTCCTT
GSH1_4	GACGTTTGCCATGACAAGATATTAACTGAGCTTAATAT
GSH1_5	AGCGGAAGATCTTTAATGTTAATAAAGTCGGGGC
GSH1_6	CATTAAGGATCCGTGGAATCAT
GSH1_7	GCCCATGCCAAAACCCATAGAATCCATATAAATGAAAC
GSH1_8	CTATGGGTTTTGGCATGGGC
GSH1_9	GATATCCCCGGGGCCTTATCATTCTCTAATAGTCTTCCTAATACTTTTTCATT
GSH1_10	GATATCAATATTGGACTATGATCTTGCTAAACATTTTGCGCATTTGTACATAAGAGATC
GSH1_11	CACTTCAAATGGTCTGAACTCCACTCTCCAACCAGGAG
GSH1_12	AGTTCAGACCATTTGAAGTG
GSH1_13	TTCCCATACTTTGGACATGTGAATATATGCGTTGATATTATCGGAAAAGGTC
GSH1_14	CACATGTCCAAAGTATGGGAA
GSH1_15	TCTAGAACTAGCTTAACATTTGCTTTCTATTGAAG
GSH1_16	CAATCACCGTGTCACCCAAATCG

Table S4. Primers used for cloning of *S. cerevisiae PRO1* gene.

Primers	Oligonucleotide sequences (5'-3')
Pro1_1	GGTCATTTTATCAGCTACTGTAATATAG
Pro1_2	AAAGGATCCATATGAAGGATGCTAATGAGAGTAAATC
Pro1_3[a]	GAATTCATGGATCCGCCGCCACCGCCACCACGAGGTGG GAATGCCAAATTTTC
Pro1_4	AAGAAACAGCTGCTCTGCACAATTCTTC
Pro1_5	TTCGTTGGCTAGCTCAACGAGGTGGGAATGCCAAATTT

[a] Nucleotide sequence encoding the linker is underlined.

Table S5. Primers used for cloning of *E. coli proB* mutant optimized according to codon usage of *S. cerevisiae.*

Primers	Oligonucleotide sequences (5'-3')
ProB1_1	GGTATCCATATGTCAGACTCTCAGACTTTGGTGGTAAAATTAGGTACTTCAGTTTTAAC
ProB1_2	AGGTACTTCAGTTTTAACAGGCGGATCAAGACGTTTGAACAGAGCTCATATCGTTGAAT
ProB1_3	GAGCTCATATCGTTGAATTAGTTAGACAATGCGCTCAATTACATGCTGCCGGTCATAGG
ProB1_4	CATGCTGCCGGTCATAGGATTGTTATTGTGACTTCTGGCGCAATTGCAGCTGGAAGAGA
ProB1_5	AATTGCAGCTGGAAGAGAACACTTGGGTTACCCAGAATTGCCAGCTACTATCGCCTCGA
ProB1_6	TGAATCAATCTTGATTGACCTACAGCTGCCAACAATTGTTTCGAGGCGATAGTAGCTGG
ProB1_7	CGACATGAATGCCATAAATAGAAAACAACTGTTCCCACAATTGAATCAATCTTGATTGA
ProB1_8	TTCTCTGTCTTCCATATCAGCTCTGGTCAACAACATTTGACCGACATGAATGCCATAAA
ProB1_9	TCTAACAAAGCTCTCAAAATATCTCTAGCGTTCAAGAACCTTTCTCTGTCTTCCATATC
ProB1_10	TCCATGGTTAACGATATTGTTATCTAACAAAGCTCTCAA
ProB2_1	TCTAGAAGGCCTGTAATTAATGAAAACGATGCTGTTGCTACAGCAGAAATTAAGGTCGG
ProB2_2	AGCAGAAATTAAGGTCGGTGATAACGATAATTTGTCTGCTTTGGCTGCAATTTTGGCTG
ProB2_3	TGGCTGCAATTTTGGCTGGTGCTGATAAATTATTGTTATTGACCGATCAAAAAGGTTTG
ProB2_4	ACCGATCAAAAAGGTTTGTATACCGCTGATCCAAGATCAAATCCACAGGCAGAATTGAT
ProB2_5	TCCACAGGCAGAATTGATTAAAGATGTTTACGGCATTGATGACGCATTGAGAGCTATTG
ProB2_6	ACGCATTGAGAGCTATTGCTGGTGATTCTGTTTCAGGCTTAGGAACTGGTGGCATGTCA
ProB2_7	GGAACTGGTGGCATGTCAACTAAATTGCAGGCCGCTGACGTGGCTTGCCGTGCTGGTA
ProB2_8	TGGCTTGCCGTGCTGGTATCGACACTATTATTGCCGCTGGTTCTAAGCCAGGTGTTATT
ProB2_9	TCTAAGCCAGGTGTTATTGGTGATGTGATGGAAGGCATTTCTGTTGGTACGTTGTTCCA
ProB2_10	AAATCCATCTTTTTCTGTTTTCTAATGGAGTAGCCTGAGCATGGAACAACGTACCAACA
ProB2_11	CTTCATCTACAGTGATTTCACCAGCAGGTGGAGCACCGAAAATCCATCTTTTTCTGTTT
ProB2_12	CAACAAAGATGAACCTCTTTCCAAAATAGCGGCAGTTGCACCTTCATCTACAGTGATTT
ProB2_13	CCTCTTGAAAAATTACCAGTCACAGATTTAATACCTTTTGGCAACAAAGATGAACCTCT

ProB2_14	CGATGTCTCTACCTTCCAAGTTACAAATTCTAATAACTTC ACCTCTTGAAAAATTACCA
ProB2_15	TCTCCTTAATGCATCAGAATTATATCTTGAGACGCCATGA GCGATGTCTCTACCTTCCA
ProB2_16	CCCAAAATTGCATCAATTTCTTGAGAGTGATGTCCGGCA ATTCTCCTTAATGCATCAGA
ProB2_17	TCATGTCATCTCTATGAACAGCAACTGGGCCGTATTCATA TCCCAAAATTGCATCAATT
ProB2_18[a]	GAATTCATGGATCCGCCGCCACCGCCACCTCTGGTAATC ATGTCATCTCTATGAAC

[a] Nucleotide sequence encoding the linker is underlined.

Table S6. Primers used for cloning of *E. coli gshB* mutant optimized according to codon usage of *S. cerevisiae.*

Primers	Oligonucleotide sequences (5'-3')
GSHB1_1	GATATCAAGATCTATGATTAAATTGGG
GSHB1_2	AAGATCTATGATTAAATTGGGTATTGTTATGGACCCAATC GCTAATATTAATATTAAGA
GSHB1_3	CTAATATTAATATTAAGAAAGATTCTTCTTTTGCTATGTT GTTAGAAGCACAAAGAAGA
GSHB1_4	TTAGAAGCACAAAGAAGAGGTTATGAATTGCATTATATG GAAATGGGTGATTTGTATTT
GSHB1_5	AATGGGTGATTTGTATTTGATTAATGGTGAAGCTAGAGC ACATACTAGAACTTTGAATG
GSHB1_6	ATACTAGAACTTTGAATGTTAAACAAAATTATGAAGAGT GGTTTTCTTTCGTTGGTGAA
GSHB1_7	TTTTCTTTCGTTGGTGAACAAGATTTGCCATTGGCTGAT TTAGATGTTATTTTGATGAG
GSHB1_8	AGTAGCATAAATAAATTCAGTATCAAAAGGTGGATCTTT TCTCATCAAAATAACATCTA
GSHB1_9	ACAATCAAAGTACCTTTCTCTTCAGCTCTTTCCAAAATA TAAGTAGCATAAATAAATTC
GSHB1_10	ACAATTTTTCATTACAATCTCTCAAAGATTGTGGTTTATT AACAATCAAAGTACCTTTC
GSHB1_11	ACCAATGTTTCTGGAGTTAAATCAGAAAACCAAGCAGT AAACAATTTTTCATTACAATC
GSHB1_12	GTTTTTCCCAAAAAGCCTTTAATTGAGCTTTATTTCTAGT AACCAATGTTTCTGGAGTT
GSHB1_13	TCCACCCATACCATCCAATGGTTTCAAAATTATATCAGAA TGTTTTTCCCAAAAAGCCT
GSHB2_1	CCATTGGATGGTATGGGTGGAGCTTCAATTTTTAGAGTT AAAGAAGGTGATCCAAATTT
GSHB2_2	AGAAGGTGATCCAAATTTGGGTGTTATTGCTGAAACTTT GACTGAACATGGTACTAGAT
GSHB2_3	CTGAACATGGTACTAGATATTGTATGGCTCAAAATTATTT GCCAGCTATTAAAGATGGT
GSHB2_4	CCAGCTATTAAAGATGGTGATAAAAGAGTGTTGGTTGTA GATGGTGAACCAGTTCCATA
GSHB2_5	TGGTGAACCAGTTCCATATTGTTTGGCTAGAATACCACA AGGTGGAGAAACTAGAGGTA
GSHB2_6	GTGGAGAAACTAGAGGTAATTTGGCTGCCGGTGGTAGA GGTGAACCAAGACCATTGACT
GSHB2_7	AAAGTTGGACCAATTTGTCTAGCAATTTTCCAATCAGAT TCAGTCAATGGTCTTGGTTC
GSHB2_8	TATAATATCCAAACCAACAAAAATCAAACCTTTTTCTTT CAAAGTTGGACCAATTTGTC
GSHB2_9	CATGTTGGAGAAGTAACATTAATTTCAGTCAATCTATCA CCTATAATATCCAAACCAAC
GSHB2_10	CAGTAATAGAAACTGGAAATTCTGCTTCAATTTCTCTAA TACATGTTGGAGAAGTAACA

GSHB2_11	TTGCTGTTGTAATCTTGCTTCAATAGCATCCATTAACATT CCAGTAATAGAAACTGGAA
GSHB2_12	GAATTCGGCTAGCTTATTGCTGTTGTAATCTTGCTTC

Table S7. Primers used for real-time PCR to verify copy number of *gshF* gene.

Primers	Oligonucleotide sequences (5'-3')
GshF_RT1	GCCAGCAAATGTGAAAGG
GshF_RT2	TGACCATCAGCAGGAATAGA
ACT_RT1[a]	TGTGATGTCGATGTCCGTAA
ACT_RT2	AAGAAGCCAAGATAGAACCA

[a] Primers used for amplification of endogenous reference *β-actin* gene.

Table S8. Relative quantitation of copy number of *gshF* gene.

Strains	C_T of *gshF* (target)	C_T of *β-actin* (reference)	relative copy number of *gshF*
W303-1b/F (positive control)	14.97	15.09	1
W303-1b/FF 19#	15.83	17.06	2.16±0.05
W303-1b/FF 24#	14.24	15.38	2.01±0.07
W303-1b/FF 33#	13.86	15.04	2.09±0.04

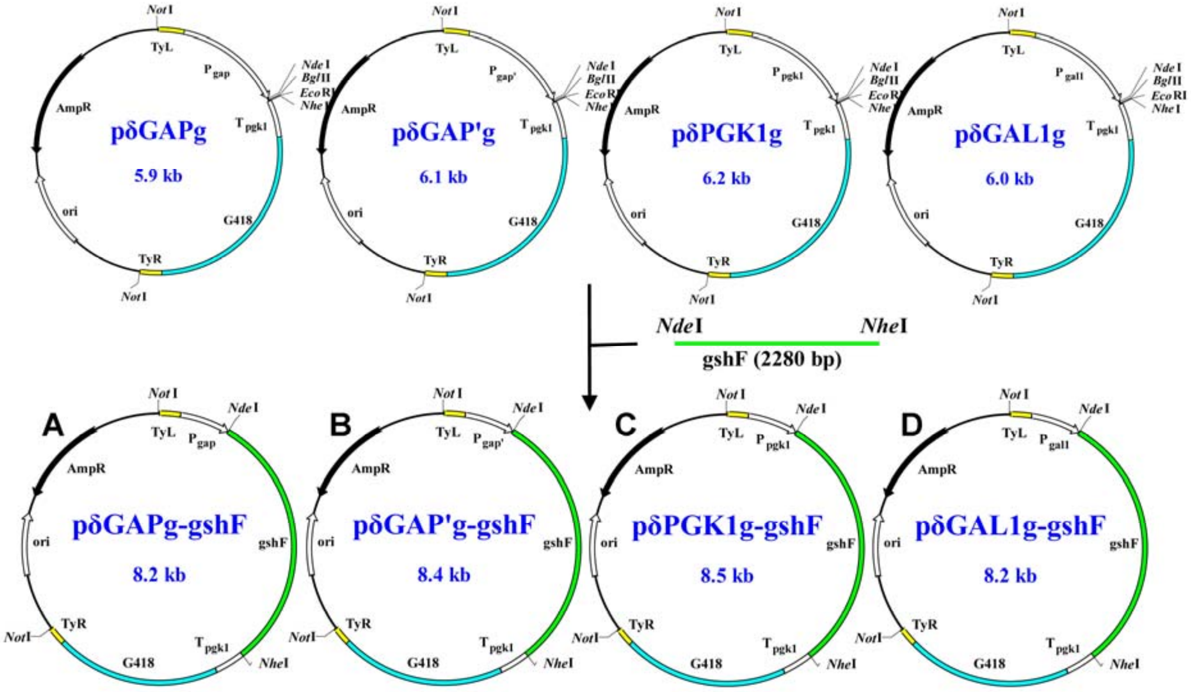

Figure S1. Construction of integrative expression vectors pδGAPg-gshF, pδGAP'g-gshF, pδGAL1g-gshF and pδPGK1g-gshF used to evaluate effects of different promoter strengths on the activity of GshF. The DNA fragment (2280 bp) of *gshF* gene obtained by double restriction digestions of pUC19-GshF with *Nde*I and *Nhe*I was inserted into plasmid pδGAPg constructed previously in our lab, generating the expression vector pδGAPg-gshF (A). Following the construction of plasmid pδGAPg-gshF, the expression vectors pδGAP'g-gshF (B), pδPGK1g-gshF (C) and pδGAL1g-gshF (D) with different promoters were constructed.

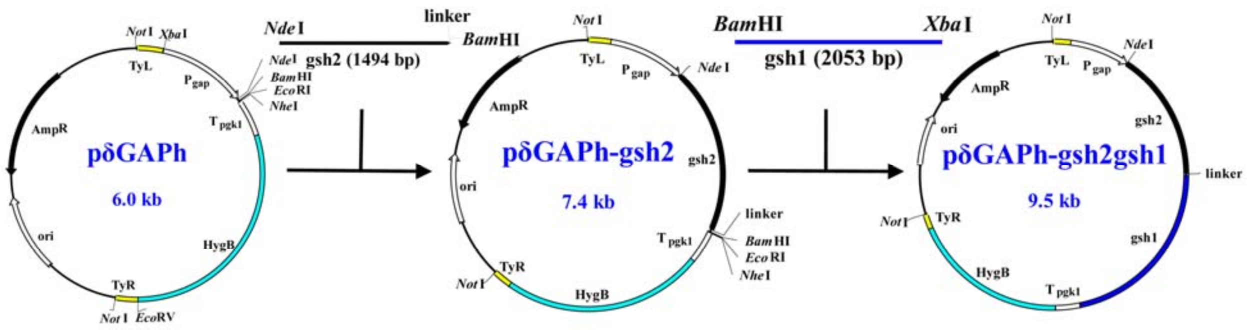

Figure S2. Construction of integrative expression vector pδGAPh-gsh2gsh1 for overexpression of Gsh2-Gsh1 fusion protein. The plasmid pδGAPh-gsh2 was generated by subcloning of *GSH2* DNA fragment (1494 bp) in plasmid EZ-GSH2 into plasmid pδGAPh constructed previously in our lab. The *GSH1* DNA fragment (2053 bp) obtained by double digestions of EZ-GSH1 with *Bam*HI and *Xba*I was ligated into plasmid pδGAPh-gsh2 digested by *Bam*HI and *Nhe*I to create the expression vector pδGAPh-gsh2gsh1.

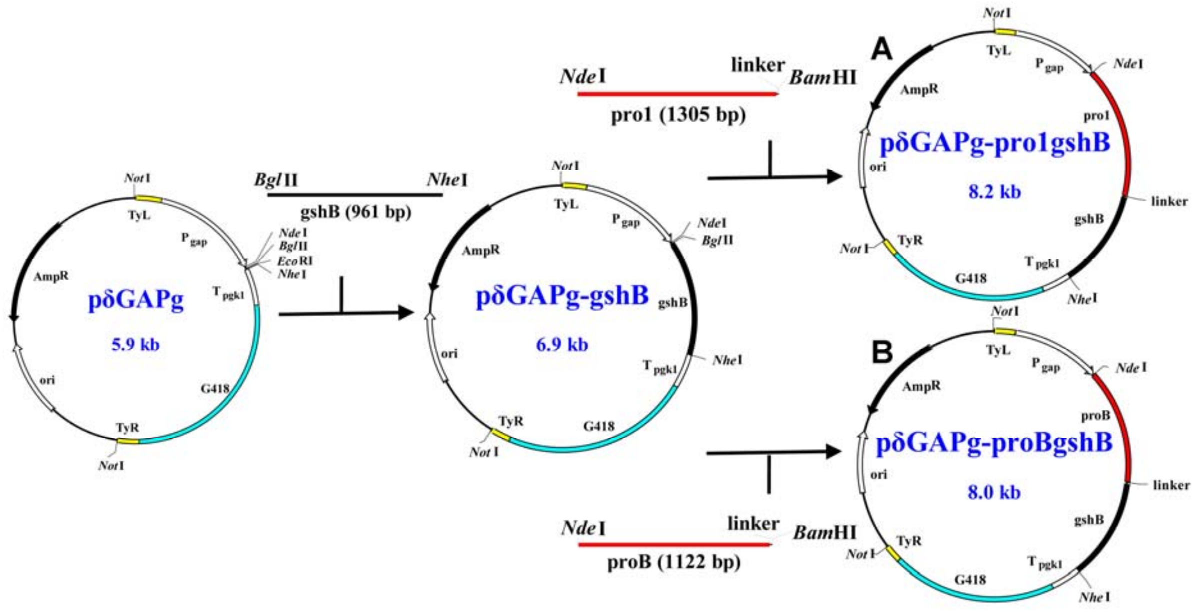

Figure S3. Construction of integrative expression vectors pδGAPg-pro1gshB and pδGAPg-proBgshB for overexpression of Pro1-GshB and ProB-GshB fusion proteins. The intermediate plasmid pδGAPg-gshB was firstly generated by subcloning of *gshB* DNA fragment (961 bp) in plasmid EZ-GSHB into plasmid pδGAPg constructed previously in our lab. Two DNA fragments, the *PRO1* DNA fragment (1305 bp) amplified by PCR and the *proB* DNA fragment (1122 bp) in plasmid EZ-PROB, were cleaved by restriction enzymes *Nde*I and *Bam*HI and inserted into plasmid pδGAPg-gshB digested by *Nde*I and *Bgl*II to generate the expression vector pδGAPg-pro1gshB (A) and pδGAPg-proBgshB (B), respectively.

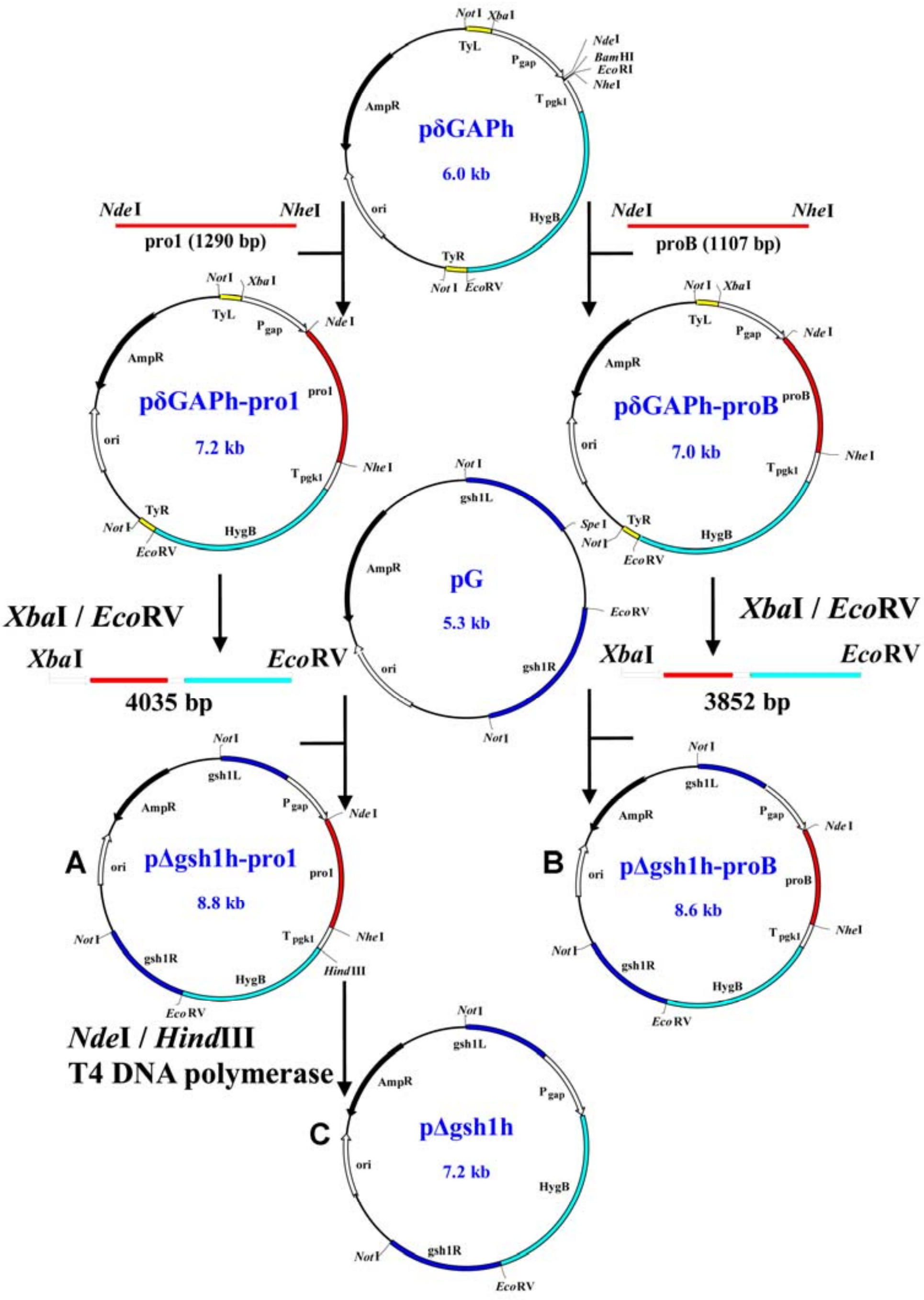

Figure S4. Construction of plasmids pΔgsh1h-pro1, pΔgsh1h-proB and pΔgsh1h used for deletion of *GSH1* gene. (A) The *PRO1* DNA fragment (1290 bp) was amplified by PCR with a pair of primers Pro1_2 and Pro1_5 (TableS4) for introducing *Nde*I and *Nhe*I sites and plasmid template of pδGAPg-pro1gshB, and then inserted into plasmid pδGAPh to generate plasmid pδGAPh-pro1. The recombinant construct of PRO1 (4035 bp) containing GAP promoter, PGK1 terminator and selective marker HygB was obtained by double digestions of plasmid pδGAPh-pro1 with *Xba*I and *Eco*RV, and directly cloned into plasmid pG

constructed previously at the *Spe*I-*Eco*RV sites, yielding plasmid pΔgsh1h-pro1 for replacement of *GSH1* by *PRO1*. (B) The plasmid pΔgsh1h-proB for replacement of *GSH1* by *proB* was created as constructed pΔgsh1h-pro1. (C) The plasmid pΔgsh1h was derived from the self-ligation of plasmid pΔgsh1h-pro1 digested with *Nde*I and *Hind*III and then filled in with T4 DNA polymerase.

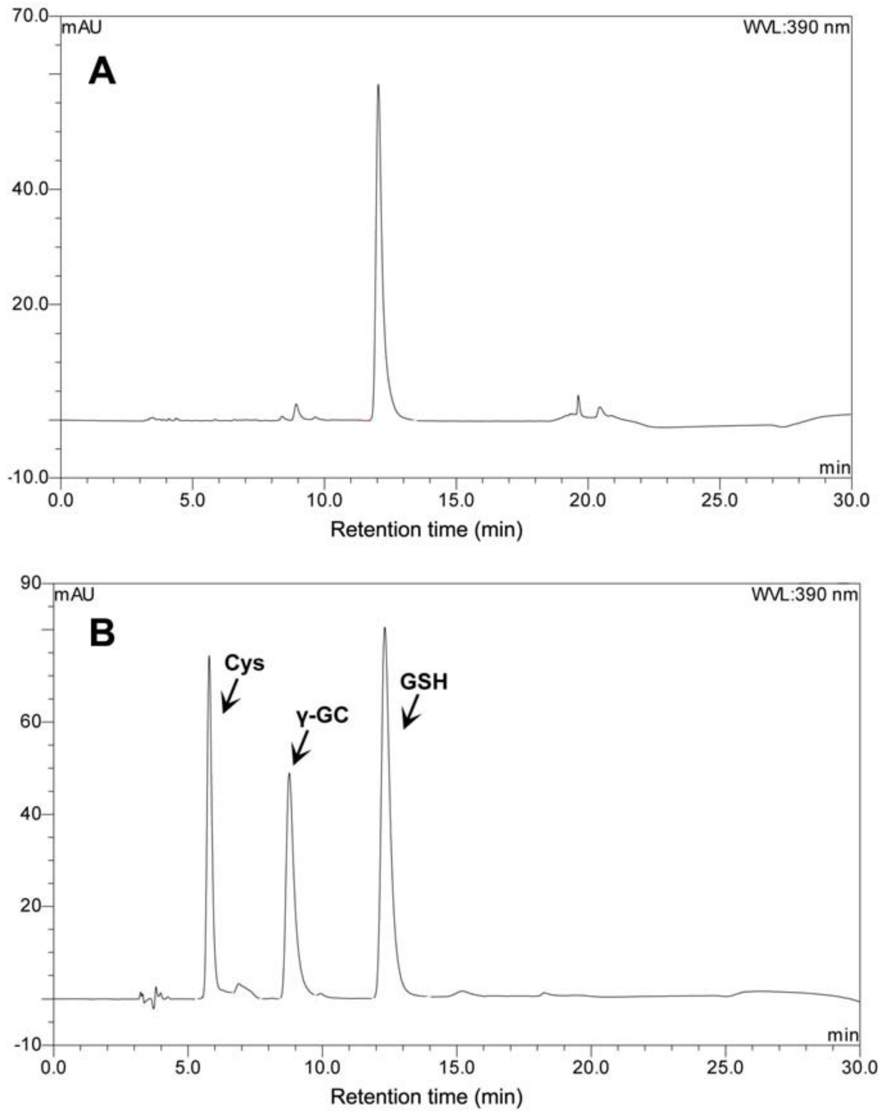

Figure S5. Chromatograms of standard thiols and samples labelled with ABD-F. (A) Intracellular thiols of engineered strain W303-1b/FGP. (B) Thiol standards of Cys, γ-GC and GSH.

Methods

1. Cloning of related genes used in this work

Cloning of *A. pleuropneumoniae gshF* gene optimized according to codon usage of *S. cerevisiae*

To functionally express heterologous *A. pleuropneumoniae gshF* (GenBank No.: **GU138097**) in *S. cerevisiae*, the codon-conformed *gshF* DNA fragment was resynthesized through splice overlap extension PCR (SOE-PCR) with the synthesized primers (Table S1). The whole DNA fragment was divided into three parts used to assemble the open reading frame (ORF) of *gshF*.

5'-terminal DNA fragment APF1 of *gshF* was obtained by SOE-PCR with primers from APF1_1 to APF1_18. Primers APF1_9 and APF1_10 were used to run a complementary reaction by PCR. Then, the fragment APF1 of the expected length (740 bp) was further obtained by eight-round SOE-PCRs with the subsequent pairs of primers APF1_8/APF1_11, APF1_7/APF1_12, APF1_6/APF1_13, APF1_5/APF1_14, APF1_4/APF1_15, APF1_3/APF1_16, APF1_2/APF1_17 and APF1_1/APF1_18. Fragment APF1 was cloned into a T-vector EZ-T (derivative of pBluescript II KS(+), GenStar, China) via TA cloning to give EZ-APF1 and verified by DNA sequencing. Middle *gshF* DNA fragment APF2 (870 bp) in plasmid EZ-APF2 and 3'-terminal *gshF* DNA fragment APF3 (709 bp) in EZ-APF3 were synthesized as amplified fragment APF1. After that, fragment APF1 obtained by digestion of EZ-APF1 with *Nde*I and *Eco*RI was inserted into plasmid pUC19, generating pUC19-APF1. Fragment APF2 isolated by digestion of EZ-APF2 with *Xho*I and *Hind*III was inserted into the *Sal*I-*Hind*III sites of plasmid pUC19-APF1 to yield pUC19-APF1-APF2. Then APF3 obtained by digestion of plasmid EZ-APF3 with *Ssp*I and *Hind*III was ligated into plasmid pUC19-APF1-APF2 digested with *Hpa*I and *Hind*III to create plasmid pUC19-GshF with a complete ORF of *A. pleuropneumoniae gshF*.

Cloning of *S. cerevisiae GSH2* encoding glutathione synthetase

To remove restriction sites *Hind*III, *Kpn*I and *Spe*I in DNA sequence of *GSH2* and sub-clone conveniently for the construction of recombinant *GSH2-GSH1* gene, primers (Table S2) were designed and synthesized according to the nucleotide sequence of *S. cerevisiae* S288C *GSH2* (GenBank No.: **NC_001147**). The whole DNA fragment was broken into three parts used to form the ORF of *GSH2*.

5'-terminal DNA fragment F1 of *GSH2* was amplified by Nested-PCR. Primers GSH2_1 and GSH2_2 were used for the first-step amplification with the genomic DNA of *S. cerevisiae* W303-1b, then fragment F1 of the expected size (665 bp) was obtained by Nested-PCR from the first-round PCR product with primers GSH2_3 and GSH2_4. Fragment F1 was cloned into a T-vector via TA cloning to give EZ-Gsh2-F1 and verified by DNA sequencing.

Middle DNA fragment F2 of *GSH2* was obtained by SOE-PCR with primers from GSH2_5 to GSH2_12. The primers GSH2_8 and GSH2_9 were used to run a complementary reaction by PCR. Then fragment F2 of *GSH2* of the expected length (228 bp) was further synthesized by three-round SOE-PCRs with the subsequent pairs of primers GSH2_7/GSH2_10, GSH2_6/GSH2_11 and GSH2_5/GSH2_12.

3'-terminal DNA fragment F3 of *GSH2* was obtained through Nested-PCR and SOE-PCR. Primers GSH2_1 and GSH2_2 were used for the first-step amplification with the genomic DNA of *S. cerevisiae* W303-1b, then two fragments of the expected size (199 bp and 391 bp) were amplified by Nested-PCR from the first-round PCR product using two pairs of primers GSH2_13/GSH2_14 and GSH2_15/GSH2_16. A further complementary reaction between 199 bp and 391 bp fragments was carried out. Then, fragment F3 of the expected length (590 bp) was amplified by SOE-PCR with primers GSH2_13 and GSH2_16 from the PCR product of complementary reaction. At last, the recombinant fragment F23 (864 bp) was generated by SOE-PCR from the PCR product of the complementary reaction between two fragments F2 and F3 with primers GSH2_5 and GSH2_16, in which an in-frame sequence encoding six-glycine linker was incorporated into the 3' terminal of *GSH2* during the PCR reaction.

The resulting fragment F23 was cloned into a T-vector via TA cloning to generate EZ-GSH2-F23 and confirmed by DNA sequencing. The plasmid EZ-GSH2 with the complete *GSH2* ORF was obtained by ligation of fragment F1 in EZ-GSH2-F1 cleaved by *Eco*RV and *Spe*I into plasmid EZ-GSH2-F23.

Cloning of *S. cerevisiae GSH1* encoding γ-glutamylcysteine synthetase

Sixteen primers (Table S3) derived from the nucleotide sequence of *S. cerevisiae* S288C *GSH1* (GenBank No.: **X85021**) were designed and synthesized. The whole DNA fragment was divided into three parts for re-assembling the complete *GSH1* ORF in order to eliminate restriction sites of *Bam*HI, *Eco*RI, *Nco*I, *Nde*I and *Ssp*I and

conveniently construct recombinant *GSH2-GSH1* gene.

5'-terminal DNA fragment F1 of *GSH1* was obtained by Nested-PCR. Primers GSH1_1 and GSH1_16 were used to run the first-step amplification with the genomic DNA of *S. cerevisiae* W303-1b, then two fragments of the expected length (231 bp and 323 bp) were amplified by Nested-PCR with the two pairs of primers GSH1_2/GSH1_3 and GSH1_4/GSH1_5. A complementary reaction between 231 bp and 323 bp fragments was carried out. Then, fragment F1 of the expected length (536 bp) was obtained by SOE-PCR with the primers GSH1_2 and GSH1_5 from the PCR product of the complementary reaction, and cloned into a T-vector via TA cloning to generate EZ-GSH1-F1 for verifying by DNA sequencing.

Middle DNA fragment F2 (705bp) of *GSH1* in the plasmid EZ-GSH1-F2 was created through Nested-PCR and SOE-PCR with the two pairs of primers GSH1_6/GSH1_7 and GSH1_8/GSH1_9 as amplified 5'-terminal DNA fragment F1 of *GSH1*.

The procedure for cloning 3'-terminal DNA fragment F3 of *GSH1* was different from that of middle DNA fragment F2. Three intermediate fragments (235 bp, 137 bp and 442 bp) were amplified by PCR using the three pairs of primers GSH1_10/GSH1_11, GSH1_12/GSH1_13 and GSH1_14/GSH1_15 and then used to carry out the complementary reaction. The resulting fragment F3 of the expected length (853 bp) was amplified by SOE-PCR with the primers GSH1_10/GSH1_15 and the PCR product of the complementary reaction. Plasmid EZ-GSH1-F3 was generated by TA cloning and verified by DNA sequencing.

The complete ORF of *GSH1* in plasmid EZ-GSH1 was obtained by two consecutive DNA ligation that the fragment F3 (847 bp) isolated by digestion of EZ-GSH1-F3 with *Ssp*I and *Xba*I was inserted into plasmid EZ-GSH1-F2 cleaved by *Sma*I and *Xba*I, and the fragment F1 (523 bp) in plasmid EZ-GSH1-F1 with the *Eco*RI-*Bgl*II digestion was ligated into the intermediate EZ-GSH1-F23 digested by *Bam*HI and *Eco*RI.

Cloning of *S. cerevisiae PRO1* encoding γ-glutamyl kinase

According to the nucleotide sequence of *S. cerevisiae* S288C *PRO1* (GenBank No.: **M85293**), four primers from Pro1_1 to Pro1_4 (Table S4) were synthesized and used to clone the *PRO1* gene. The complete *PRO1* ORF (1284 bp) was amplified by Nested-PCR with the genomic DNA of *S. cerevisiae* W303-1b, and an in-frame

sequence encoding six-glycine linker was added into the 3' terminal of *PRO1* during the PCR reaction.

Cloning of *E. coli proB* mutant optimized according to codon usage of *S. cerevisiae*

On the basis of the nucleotide sequence of *E. coli* BL21 *proB* (GenBank No.: **AM946981**), the synthesized primers (Table S5) were used to clone the whole DNA fragment of *proB* mutant which was divided into two parts for DNA fragment assembly.

5'-terminal DNA fragment proB1 of *proB* mutant was amplified by SOE-PCR with primers from ProB1_1 to ProB1_10. Primers ProB1_5 and ProB1_6 were used to run a complementary reaction by PCR. Then, fragment proB1 of the expected length (408 bp) was obtained by four-round SOE-PCRs with the subsequent pairs of primers ProB1_4/ProB1_7, ProB1_3/ProB1_8, ProB1_2/ProB1_9 and ProB1_1/ProB1_10, and cloned into a T-vector via TA cloning to generate plasmid EZ-ProB1 for verifying by DNA sequencing.

3'-terminal DNA fragment proB2 (735 bp) of *proB* mutant was synthesized as amplified fragment proB1. Using the primer ProB2_18, an in-frame sequence encoding six-glycine linker was added into the 3' terminal of *proB* during the PCR reaction. The resulting plasmid EZ-ProB2 was verified by DNA sequencing after TA cloning. At last, fragment proB1 obtained by digestion of plasmid EZ-ProB1 with *Hind*III and *Hpa*I was inserted into plasmid EZ-ProB2 digested by *Hind*III and *Stu*I, generating plasmid EZ-ProB used for the construction of recombinant *proB-gshB* gene.

Cloning of *E. coli gshB* mutant optimized according to codon usage of *S. cerevisiae*

Based on the encoding sequence of *E. coli* BL21 *gshB* (GenBank No.: **NP_417422**), the synthesized primers (Table S6) were used to clone the whole DNA fragment of *gshB* mutant which was divided into two parts for DNA fragment assembly.

5'-terminal DNA fragment gshB1 of *gshB* mutant was obtained by SOE-PCR with primers from GSHB1_2 to GSHB1_13. Primers GSHB1_7 and GSHB1_8 were used for a complementary reaction by PCR. Then, the fragment gshB1 of the expected length (508 bp) was synthesized by five-round SOE-PCRs with the subsequent pairs

of primers GSHB1_6/GSHB1_9, GSHB 1_5/GSHB 1_10, GSHB1_4/GSHB1_11, GSHB1_3/GSHB1_12 and GSHB1_2/GSHB1_13.

3'-terminal DNA fragment gshB2 (484 bp) of *gshB* mutant was constructed as amplified fragment gshB1. And then, two fragments gshB1 and gshB2 were carried out a complementary reaction by PCR. At last, the whole *gshB* ORF (977 bp) was amplified by SOE-PCR with the primers GSHB1_1/GSHB2_12 and the PCR product of complementary reaction. The resulting plasmid EZ-GshB was used for DNA sequencing and further construction of expression vector.

2. GSH production of the engineered strains harbouring *gshF* gene under the control of four different promoters

Three positive colonies of each engineered strain of W303-1b/F/GAP, W303-1b/F/GAP', W303-1b/F/GAL1 and W303-1b/F/PGK1 was cultured at 30°C in shake flasks containing 20 mL of liquid YPD medium with agitation at 250 rpm for 18-24 h. An adequate volume of fresh cultures were then inoculated into a set of 250 mL flasks containing 50 mL of liquid YPD medium (W303-1b/F/GAP, W303-1b/F/GAP' and W303-1b/F/PGK1) or YPGal medium (10 g/L yeast extract, 20 g/L tryptone and 20 g/L galactose, W303-1b/F/GAL1) to keep an initial optical density at 600 nm (OD600) value of 0.2. The cultures were incubated at 30°C with agitation at 250 rpm for additional 96 h. Intracellular GSH amount was monitored after 24 hours fermentation and measured every 12 hours.
